# Excitation Energy Transfer and Exchange‐Mediated Quartet State Formation in Porphyrin‐Trityl Systems

**DOI:** 10.1002/chem.202002805

**Published:** 2020-12-01

**Authors:** Oliver Nolden, Nico Fleck, Emmaline R. Lorenzo, Michael R. Wasielewski, Olav Schiemann, Peter Gilch, Sabine Richert

**Affiliations:** ^1^ Institute of Physical Chemistry Heinrich Heine University Düsseldorf Universitätsstraße 1 40225 Düsseldorf Germany; ^2^ Institute of Physical and Theoretical Chemistry University of Bonn Wegelerstraße 12 53115 Bonn Germany; ^3^ Department of Chemistry Northwestern University 2145 Sheridan Road Evanston IL 60208-3113 USA; ^4^ Institute of Physical Chemistry University of Freiburg Albertstraße 21 79104 Freiburg Germany

**Keywords:** enhanced intersystem crossing, excitation energy transfer, excited multi-spin systems, quartet state formation, transient EPR spectroscopy

## Abstract

Photogenerated multi‐spin systems hold great promise for a range of technological applications in various fields, including molecular spintronics and artificial photosynthesis. However, the further development of these applications, via targeted design of materials with specific magnetic properties, currently still suffers from a lack of understanding of the factors influencing the underlying excited state dynamics and mechanisms on a molecular level. In particular, systematic studies, making use of different techniques to obtain complementary information, are largely missing. This work investigates the photophysics and magnetic properties of a series of three covalently‐linked porphyrin‐trityl compounds, bridged by a phenyl spacer. By combining the results from femtosecond transient absorption and electron paramagnetic resonance spectroscopies, we determine the efficiencies of the competing excited state reaction pathways and characterise the magnetic properties of the individual spin states, formed by the interaction between the chromophore triplet and the stable radical. The differences observed for the three investigated compounds are rationalised in the context of available theoretical models and the implications of the results of this study for the design of a molecular system with an improved intersystem crossing efficiency are discussed.

## Graphical abstract

**Photogenerated multi‐spin systems** composed of a porphyrin chromophore and a trityl radical are investigated with respect to their optical and magnetic properties. Profiting from the time‐resolution of fs‐TA spectroscopy and the capability of EPR to distinguish near‐degenerate states with different spin multiplicity, this work reports unique insight into the excited‐state reaction mechanism and suggests improvements to the molecular design in view of applications in the fields of molecular spintronics and artificial photosynthesis.

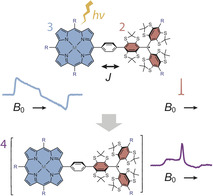

## Introduction

Due to their versatility, covalently‐linked chromophore‐radical systems have found a wide range of applications in the fields of information technology, artificial photosynthesis, as well as spin catalysis.[^1^, ^2^, ^3^, ^4^] In all of these applications, the covalent linkage of a stable radical to the chromophore is used as a means of altering—and thereby controlling—the excited state dynamics of the chromophore, and many of them rely on the ability of the stable radical to act as an efficient triplet sensitiser by enhancing the intersystem crossing rate constant.

An increased triplet yield can for instance serve to improve the efficiency of processes such as triplet‐triplet annihilation photon‐upconversion,[^5^, ^6^, ^7^] while other applications in organic solar cells or OLEDs make use of the photoluminescence (doublet emission) of a particular class of these *π*‐radical systems.[^3^, ^8^, ^9^, ^10^]

In the area of molecular spintronics, photogenerated organic multi‐spin systems have proven invaluable for exploring the fundamental requirements for spin‐information transfer and storage on a molecular level.[^11^, ^12^, ^13^, ^14^] By photoexcitation, well‐defined initial spin states with strong spin polarisation can be generated, which may then be characterised by advanced magnetic resonance techniques with regard to their magnetic properties and spin‐spin interactions. Through systematic variations of the molecular design and a careful study of the resulting magnetic properties, it will ultimately be possible to establish design protocols for new magnetic materials exhibiting the desired properties for an efficient transfer and storage of spin information.

Motivated by the large number and potential impact of these applications, a multitude of recent studies have focused on increasing our understanding of the excited state dynamics and deactivation mechanisms in chromophore‐radical systems, with the long‐term goals of achieving photocontrol of the magnetic properties of materials via spin coupling and exploring the influence of molecular topology on the interaction efficiency.[^1^, ^2^, ^3^, ^15^, ^16^, ^17^, ^18^, ^19^, ^20^, ^21^, ^22^, ^23^]

However, despite this effort, the mechanistic behaviour of organic multi‐spin systems is up to now only poorly understood and truly systematic studies that could help to elucidate the details of the excited state dynamics, the mechanisms of excited multiplet generation and spin‐information transfer, as well as the role of the individual interacting building blocks (i.e. chromophore triplet, bridge, stable radical) remain scarce.

**Figure 1:**
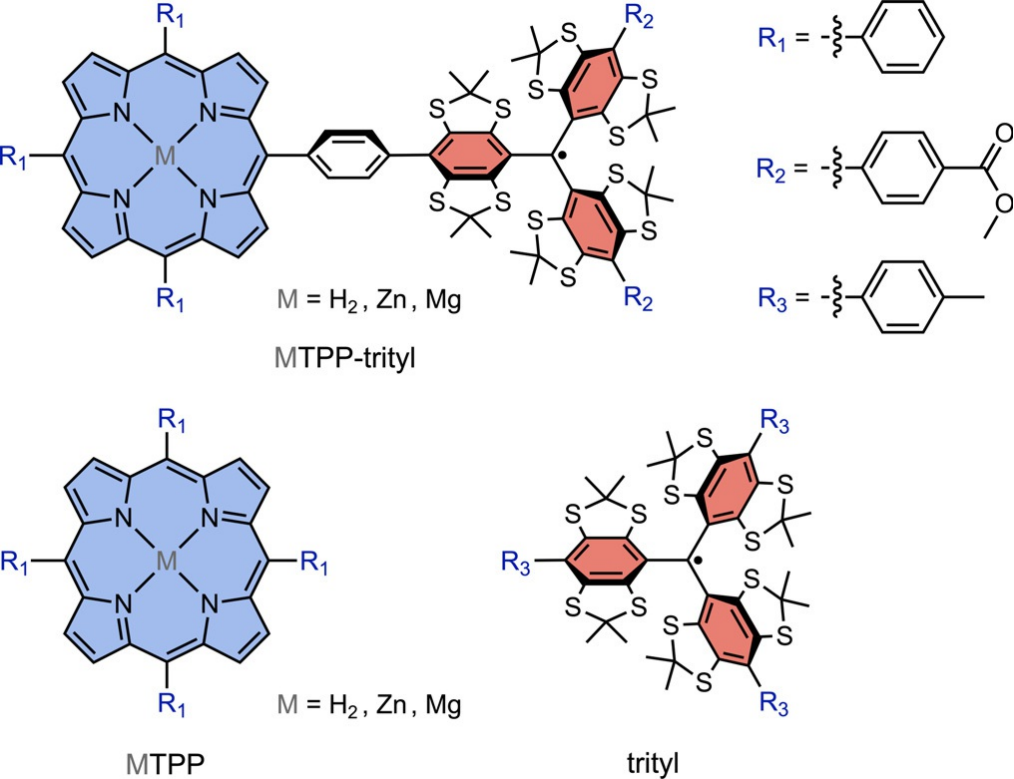
Chemical structures of the investigated chromophore‐radical systems and their building blocks.

Here, we investigate the spectroscopic properties of a series of covalently‐linked chromophore‐radical systems using femtosecond transient UV‐vis absorption (fs‐TA) and electron paramagnetic resonance (EPR) techniques. Porphyrins were chosen as the chromophores since they are highly photostable,^[24]^ exhibit characteristic EPR signatures,^[25]^ and their central metal can easily be modified, enabling a variation of the excited state energetics while avoiding major changes to the molecular structure. A tetrathiaryl trityl radical, derivatives of which are commonly employed as spin labels,[^26^, ^27^, ^28^, ^29^] was attached to the porphyrin via a phenyl linker and is used here as the stable radical moiety. Compared to more commonly employed stable radicals, such as nitroxides, trityl radicals are characterised by a very narrow EPR line and slow spin‐lattice relaxation,^[30]^ which might be particularly interesting for spin information storage applications. In addition, the chosen molecular design and synthetic approach allows a systematic modification of the individual building blocks of this photogenerated three‐spin system.^[31]^ The three investigated structures only differ with respect to the central metal bound by the tetraphenylporphyrin (TPP) chromophore as shown in Figure 1 and are henceforth referred to as H_2_TPP‐trityl, ZnTPP‐trityl, and MgTPP‐trityl.

Making use of the time‐resolution of fs‐TA spectroscopy, we explore the mechanism underlying excited multiplet generation in the porphyrin‐trityl systems and determine the efficiencies of the various competing excited state reactions. Complementary information is obtained from transient continuous wave EPR experiments, allowing us to identify and characterise the different spin states and spin–spin interactions. It is found that the interaction between the porphyrin triplet and the radical falls within the strong coupling regime, resulting in the formation of (doublet and) quartet states at cryogenic temperatures. Optical spectroscopy reveals that ultrafast energy transfer (∼10 ps) from the porphyrin to the trityl radical largely dominates the excited state dynamics at room temperature and in frozen solution, implying a very low quartet formation efficiency. However, despite this low yield, transient EPR enabled the determination of the characteristic magnetic parameters and spin polarisation patterns of the formed quartet states and provided further information on the mechanistic details of quartet formation, as well as on the internal dynamics of the spin system. Finally, the differences observed for the three dyads containing different porphyrin central metals are discussed, together with the implications of the results of this study for the design of a molecular system with an improved intersystem crossing efficiency.

**Figure 2:**
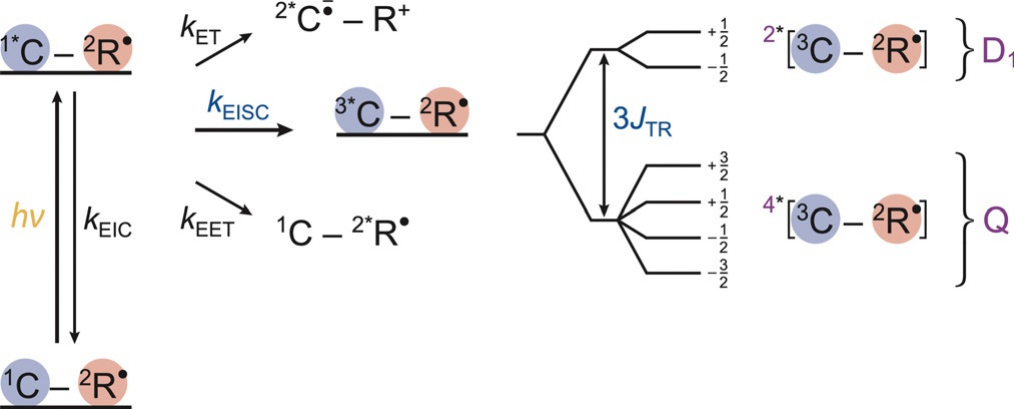
Schematic overview of the photophysical processes expected to occur in covalently‐linked chromophore‐radical systems assuming a non‐negligible exchange interaction *J*_TR_. Abbreviations: EIC enhanced internal conversion; ET electron transfer; EET excitation energy transfer; EISC enhanced intersystem crossing.

In all transient spectroscopic experiments, the porphyrins were excited at a wavelength of 550 nm, corresponding to an absorption maximum within the porphyrin Q‐band region (S_1_ state). After photoexcitation of the porphyrin chromophore, a number of different excited state reactions can occur, as schematically shown in Figure 2, mediated by the close proximity of the third spin of the stable radical. The possible processes are (i) excited state electron transfer (ET), (ii) enhanced intersystem crossing (EISC), (iii) excitation energy transfer (EET) and (iv) enhanced internal conversion (EIC) back to the ground state.

## Results and Discussion

**Synthesis and characterisation**: H_2_TPP‐trityl and ZnTPP‐trityl were synthesised according to procedures reported previously,^[31]^ while the synthesis and characterisation of MgTPP‐trityl is outlined in the Supporting Information. An NMR characterisation of the compounds and precursor molecules (cf. Figure S1) revealed that MgTPP‐trityl is prone to oxidation during the purification process. The batch of MgTPP‐trityl thus contained some diamagnetic impurity (∼30 %), where the radical centre had been transformed into the corresponding triphenylmethanol moiety. Although no such observation was made for ZnTPP‐trityl and H_2_TPP‐trityl, the presence of very small amounts (∼0–3 %) of a diamagnetic impurity in the samples cannot be completely excluded due to the limited sensitivity of ^1^H NMR, especially with respect to the radical content of the sample. The presence of such an impurity would be observed as a triplet contribution in the spectroscopic studies, but is by no means expected to influence the photophysical behaviour of the chromophore‐radical compounds.

**UV‐vis absorption spectroscopy**: The room temperature UV‐vis spectra of the compounds and their porphyrin and trityl building blocks are shown in Figures S4 and S5. As is well‐known from the literature,^[24]^ the spectra of the three tetraphenylporphyrins are qualitatively similar, showing an intense absorption band around 420 nm (porphyrin Soret band, S_2_ state) and significantly less intense absorption peaks in the wavelength region of the porphyrin Q‐bands from roughly 500 to 620 nm. The individual peak positions, however, differ for the different porphyrins and are also influenced by the solvent polarity. While the spectra of ZnTPP and MgTPP (approximate D_4h_ symmetry) show one prominent absorption peak in the Q‐band region, this Q‐band peak is shifted to higher wavelengths and split into two major peaks in H_2_TPP, due to symmetry breaking induced by the presence of the two protons.

The spectrum of the trityl radical is characterised by a very prominent absorption band in the visible at 460 nm, a strong UV absorption, and a very broad but less intense absorption band extending over the whole visible range into the NIR (∼900 nm).^[32]^ The absorption spectra of the porphyrin‐trityl compounds are similar to the sum of the UV‐vis spectra of their respective building blocks as can be seen from Figures S4 and S5 in the Supporting Information.

**Femtosecond transient absorption**: In order to get an idea about the efficiencies associated with the individual competing excited state reactions and their temperature dependence, femtosecond transient absorption measurements of the porphyrin‐trityl compounds as well as their building blocks were performed in toluene at room temperature and in isotropic frozen 2‐methyltetrahydrofuran solution at 85 K. Details on the sample preparation, experimental setup^[33]^ and data treatment can be found in the Supporting Information. Since the observations for the investigated porphyrin‐trityl compounds were qualitatively very similar, only the results obtained for ZnTPP‐trityl and its building blocks are presented in the main text, while the corresponding data for the free base analogue are shown in the Supporting Information (Figure S6).

**Figure 3:**
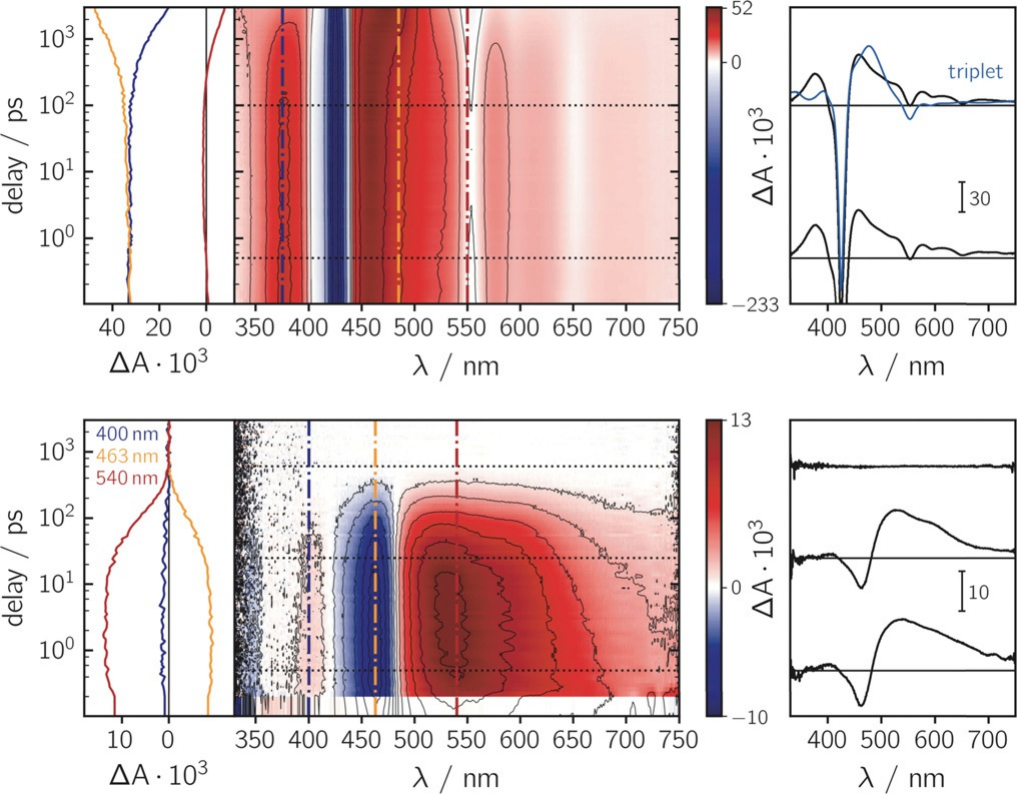
Femtosecond transient absorption data for ZnTPP (top) and the trityl radical (bottom) recorded in toluene solution at room temperature after photoexcitation at 550 nm. The central panel shows a contour plot of the data, where the red and blue colour coding represents positive and negative signals, respectively. The vertical coloured lines in the contour plot indicate the positions corresponding to the kinetic traces shown in the left panel, while the dotted horizontal lines indicate the time delays associated with the spectra shown on the right. The spectrum overlaid in blue corresponds to the spectrum of the ZnTPP excited triplet state, obtained from a global kinetic analysis of the data.

Figure 3 shows the room temperature fs‐TA data of the ZnTPP chromophore and trityl radical moieties. The photophysics of ZnTPP is well‐known and has already been studied extensively.[^34^, ^35^] In agreement with these previous results, we observe a strong ground‐state bleach around 420 nm, accompanied by a considerably weaker excited state absorption extending over the entire visible range, with a maximum close to the onset of the ground state bleach at 460 nm. These typical features of the ZnTPP excited singlet state decay with a time constant of about 2.6 ns to form the porphyrin triplet state in high yield.[^36^, ^37^, ^38^] The latter then lives for about 1 μs in solution at room temperature.^[36]^

A global kinetic analysis of the data, as presented in detail in the Supporting Information, revealed that the evolution of the ZnTPP spectra with time can be described satisfactorily with two time constants of 11 ps and 2.6 ns. In addition, we detect an offset which is in line with the ∼μs lifetime of the porphyrin triplet state. The first time constant is likely to be associated with relaxation processes within the S_1_ state, while the second time constant can be assigned to the decay of the excited singlet state and simultaneous rise of the T_1_ state. As shown in Figure 3, the signatures of the porphyrin excited singlet and triplet states are very similar and may therefore be difficult to distinguish.

The fs‐TA spectra obtained for the trityl radical are shown in Figure 3 (bottom). They are characterised by a ground state bleach centred at about 460 nm, and a single, broad, and rather featureless excited state absorption extending from roughly 480 to ∼ 800 nm, with a maximum around 540 nm. The excited state absorption features do not change markedly over the course of the excited state lifetime. These features and the ground state bleach decay completely with a time constant of 120 ps, as determined by a global kinetic analysis of the data (cf. SI).

**Figure 4:**
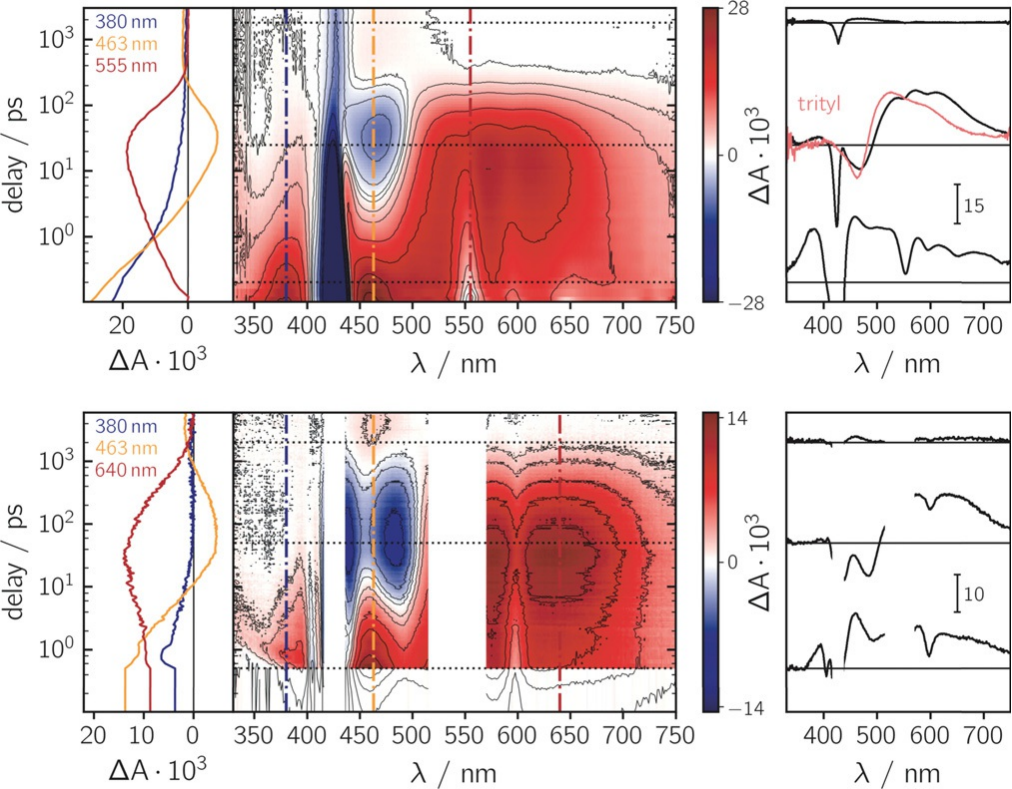
Femtosecond transient absorption data for ZnTPP‐trityl recorded in toluene solution at room temperature (top) and frozen 2‐methyltetrahydrofuran solution at 85 K (bottom) after photoexcitation at 550 nm. For a comparison of the spectral shape, a representative spectrum of the excited trityl radical is superimposed onto the ZnTPP‐trityl data (red line). The wavelength ranges dominated by artefacts from either excitation light scattering or optical saturation were cut out from the spectra recorded at 85 K. In the room temperature data, the ground state bleach extending to Δ*A*=0.22 has been truncated for a better comparability of the relative spectral signals.

In the proximity of the trityl radical, the excited state dynamics of ZnTPP are considerably altered as evident from Figure 4, showing the spectra of ZnTPP‐ trityl recorded at room temperature (top) and in frozen solution (bottom). Shortly after photoexcitation of the porphyrin moiety, the typical features of the ZnTPP singlet excited state are observed and found to decay quickly, with a time constant of ∼10 ps. The decay of the S_1_ state is accompanied by the simultaneous rise of a new absorption band in the range from 480 to 800 nm and the formation of a new ground state bleach centred at 460 nm. A global kinetic analysis of the room temperature data (cf. SI) revealed that these newly formed excited state features subsequently decay with a time constant of roughly 120 ps. After their complete decay, a small percentage (∼ 4–5 %) of the porphyrin ground state bleach remains, together with some excited state absorption, attributed to the porphyrin triplet state.

In frozen solution, the overall reaction dynamics are qualitatively similar, as shown in Figure 4 (bottom). However, the spectral positions of the individual excited state features appear to be slightly redshifted, due to the different solvent environment (e.g. dielectric constant) and temperature, and the excited state absorption is found to decay with a slower time constant of 650 ps.

The room temperature fs‐TA experiments on ZnTPP‐trityl were also performed at a different excitation wavelength (i.e. 400 nm) and in 2‐methyltetrahydrofuran solution (cf. SI). No marked differences in the spectra were observed, suggesting that the excited state deactivation mechanism in these systems is largely independent of solvent polarity and excitation wavelength (porphyrin Soret vs. Q‐band excitation). Even in polar solvents, no spectral features characteristic of ZnTPP ion formation could be observed,^[39]^ indicating that any contribution of excited state electron transfer to the excited state dynamics can likely be excluded.

When comparing the room temperature fs‐TA spectrum obtained for ZnTPP‐trityl after the initial decay of the porphyrin S_1_ state, e.g. at 25 ps, with that of the excited trityl radical (cf. Figure 4, top), a strong similarity is evident. The spectral features are nearly identical, which is a clear indication for excitation energy transfer (EET) from the porphyrin to the trityl radical taking place in ZnTPP‐trityl. A detailed analysis of the relative spectral amplitudes and time constants (cf. SI) demonstrated that this energy transfer (i) is very efficient with a quantum efficiency of *Φ*_EET_≥95 % and (ii) occurs equally fast at room temperature and in frozen solution, with a time constant of ∼10 ps (no thermal activation barrier).

For energy transfer from the chromophore to the radical to occur, the radical needs to have low‐lying electronic states that are accessible from the chromophore's excited singlet state. In principle, two different energy transfer mechanisms could be invoked to explain the experimental observations: Dexter‐type energy transfer requires orbital overlap as it is based on an electron exchange mechanism. The associated transfer rates show an exponential distance dependence and a strong dependence on the bridge structure.[^40^, ^41^] On the other hand, Förster resonance energy transfer is based on a dipolar mechanism, requiring spectral overlap of the fluorescence spectrum of the energy donor (ZnTPP) and the absorption spectrum of the energy acceptor (trityl), but no orbital overlap. The transfer rate depends on the distance between donor and acceptor as *r*_DA_^−6^.^[42]^

In order to evaluate whether the experimentally observed rate constants could be in agreement with Förster theory, we performed calculations of the expected Förster energy transfer rate for ZnTPP‐trityl, as detailed in the Supporting Information. The employed formalism relies on the validity of the point‐dipole approximation and is therefore likely not to yield accurate results in the case of the investigated structures, since the centre‐to‐centre distance between chromophore and radical is not significantly larger than their individual molecular sizes. Nevertheless, a good estimate can be obtained. The calculations were carried out with a centre‐to‐centre distance of *r*_DA_=1.3 nm and for different values of *κ* ^2^, describing the relative orientation of the transition dipole moments within the porphyrin and trityl moieties with respect to the vector connecting them. The correct value for *κ* ^2^ cannot easily be predicted, but given the covalent linkage between donor and acceptor and the known orientation of the transition dipole moments within the porphyrin and trityl moieties, it might be reasonable to assume that *κ* ^2^ is larger than one and maybe even close to its maximum value of four. Provided this assumption holds true, energy transfer time constants between 10 and 40 ps should be feasible (cf. SI).

The experimentally observed EET time constant of ∼10 ps thus seems to be consistent with a Förster‐type mechanism, although a contribution from Dexter‐type energy transfer cannot be excluded in view of the rather short distance between donor and acceptor and the covalent linkage between the two reaction partners, resulting in non‐negligible exchange coupling.^[31]^

The high efficiency of excitation energy transfer in these covalently‐linked porphyrin‐trityl systems naturally imposes severe limitations on the efficiency of the enhanced intersystem crossing (EISC) process and therefore the efficiency of excited multiplet formation. Efficient EISC should result in rapid quenching of the porphyrin S_1_ state accompanied by a simultaneous rise of the porphyrin T_1_ state. Here, we find that excited state deactivation is dominated by EET, suggesting that the rate constant of EISC is at least an order of magnitude smaller than that found for EET. From the ratio of the intensities of the initial ground state bleach and the ground state bleach remaining after complete decay of the trityl excited state absorption, we can estimate that the quantum efficiency of EISC can at most reach a value of ∼5 % in these systems.

In addition, part of this remaining triplet absorption could also result from a contribution of molecules where either (i) the trityl radical has been deactivated (diamagnetic impurity) or (ii) the orbital overlap between the chromophore and trityl moieties is negligibly small (unfavourable molecular conformation). Such molecules would undergo “normal”, i.e. spin‐orbit coupling induced, intersystem crossing (ISC) to the porphyrin triplet state.

Since different origins for the triplet state contribution to the fs‐TA spectra of ZnTPP‐trityl can be envisioned, only an upper limit for the EISC yield of *Φ*_EISC_≤5 % can be given as a result from the analysis of the femtosecond transient absorption data.

**Electron paramagnetic resonance**: In order to verify whether excited multiplet formation can be observed for the different porphyrin‐trityl compounds and to characterise the magnetic properties of the individual spin states formed by the interaction of the porphyrin triplet and the trityl radical, transient continuous wave (cw) EPR measurements were performed in isotropic frozen 2‐methyltetrahydrofuran solution at 80 K. Details on the sample preparation and experimental setup are given in the Supporting Information.

**Figure 5:**
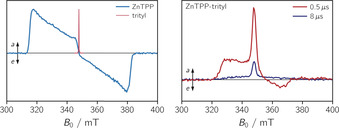
Comparison of the transient cw EPR spectrum of the triplet state of ZnTPP with the field‐swept FID‐detected spectrum of the trityl radical (left) and transient cw EPR spectra of ZnTPP‐trityl at two different time delays after photoexcitation at 550 nm (right). All spectra were recorded at the X‐band (9.75 GHz) in frozen 2‐methyltetrahydrofuran at 80 K.

Figure 5 (left) shows a comparison of the transient cw EPR spectrum of the ZnTPP triplet state and the spectrum of the trityl radical, recorded in pulse mode. The spectrum of the trityl radical is very narrow, due to the lack of hyperfine coupling interactions to nearby magnetic nuclei, and exhibits an isotropic *g* value of 2.0028, as determined by a spectral simulation of the corresponding room temperature cw EPR spectrum (cf. Figure S13). Some slight structural broadening is observed in the wings of the spectrum due to ^13^C satellite‐transitions.^[31]^

Compared to the spectrum of the trityl radical, the triplet state spectrum of ZnTPP is very broad. As it is typical for the triplet states of organic chromophores,^[43]^ the spectral shape and width is dominated by the zero‐field splitting (ZFS) interaction, described by the following Hamiltonian:

(1) $${ℋ}_{ZFS}=SDS=D\left(S_{z}^{2}-\frac{1}{3}S^{2}\right)+E\left(S_{x}^{2}-S_{y}^{2}\right)$$

and parametrised by the two ZFS parameters *D* and *E*. In the case of the investigated porphyrins, *D* is known to be positive as determined from magnetophotoselection experiments,^[44]^ implying an oblate spin density distribution. Due to differences in the intersystem crossing rates to the individual triplet sublevels, triplet spectra are typically spin‐polarised, leading to the observation of absorptive (*a*) as well as emissive (*e*) transitions.

To determine the zero‐field splitting *D* values as well as the relative initial populations of the three triplet sublevels, numerical simulations of the triplet state spectra were carried out. In good agreement with previously published results,[^45^, ^46^] *D*_T_ values of 930 MHz, 1150 MHz and 890 MHz were obtained for ZnTPP, H_2_TPP and MgTPP, respectively. While the out‐of‐plane triplet sublevel (*Z*) is primarily populated in ZnTPP, resulting in an *aaaeee* spin polarisation pattern, the in‐plane levels (*X*, *Y*) are overpopulated in H_2_TPP and MgTPP as evident from the *eeeaaa* polarisation pattern. The experimental spectra, simulations and further simulation parameters for all three tetraphenylporphyrins are shown in Figure S14 in the Supporting Information.

Compared to the spectra of the two building blocks, significant differences are observed in the transient cw EPR spectra of ZnTPP‐trityl as shown in Figure 5 (right). The overall spectral width is considerably reduced compared to that of the ZnTPP triplet state and a strong absorptive feature is found to dominate the central part of the transient spectrum. In addition, the spectral shape is found to change with time: While an overall *a/e* spin polarisation could be detected shortly after laser excitation, the spectra detected after a few microseconds are found to be entirely in absorption and are largely dominated by the central absorptive feature.^[47]^

This central feature with broad, significantly less intense, wings, observed after a few microseconds, is characteristic for a quartet state with Boltzmann population. In addition, a similar time evolution of the spin polarisation (*a*/*e* polarisation turning into net absorptive features) has been observed before for a porphyrin‐nitroxide system,[^1^, ^16^] and could be attributed to thermal equilibration of the populations within the quartet state.^[48]^ We therefore tentatively assign the spectral features observed here for ZnTPP‐trityl to the quartet state.

**Figure 6:**
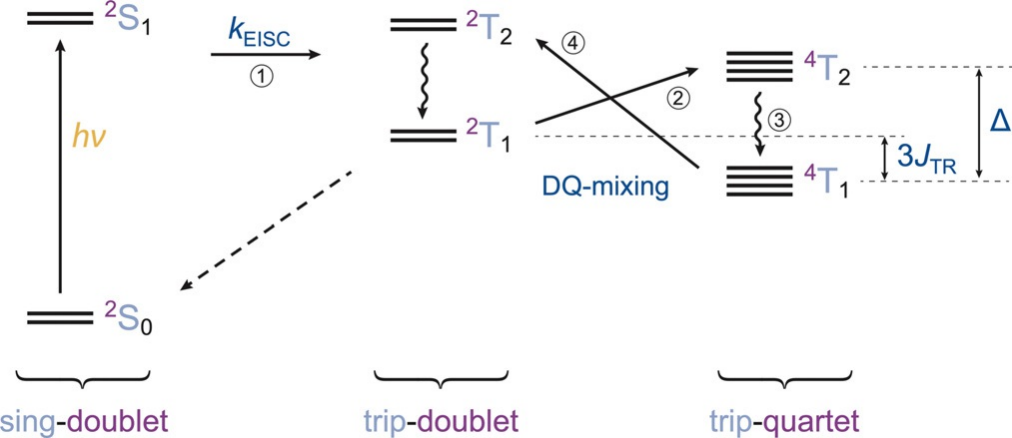
Processes and energetic states involved in quartet state formation. The individual energetic states are labelled with terms of the form ^*j*^ *A_i_*, where the superscript *j* indicates the overall spin multiplicity, *A* the spin multiplicity of the chromophore, and the subscript *i* the energetic ordering (low to high) of the states with identical multiplicities.

From a detailed analysis of the characteristic magnetic parameters (i.e. zero‐field splitting parameters *D*_Q_, *E*_Q_ and the *g* value *g*_Q_) and spin polarisation patterns of the quartet state, important information on the quartet state formation mechanism can be obtained. However, before we discuss the implications of the present experimental results, the proposed mechanism for quartet state formation,[^3^, ^18^, ^19^, ^49^] graphically summarised in Figure 6, shall briefly be outlined here.

According to this model, light absorption leads to π–π* singlet excitation of the porphyrin moiety. From this so‐called excited sing‐doublet state (^2^S_1_),^[24]^ EISC occurs to yield the chromophore triplet state. The triplet state is split into trip‐doublet (^2^T) and trip‐quartet (^4^T) states by the exchange interaction (Δ*E*_DQ_=3*J*_TR_≪*k*_B_ *T*). Typically, the lowest trip‐doublet state (^2^T_1_) is populated first by EISC, since, due to different exchange interactions of the two triplet electrons with the electron of the radical, the trip‐doublet state acquires some sing‐doublet character, implying that the relaxation from the excited sing‐doublet state to the trip‐doublet becomes partially allowed.^[24]^ The following transition from the doublet to the quartet state is spin‐forbidden, but can be mediated by spin‐orbit coupling (SOC).[^3^, ^18^, ^19^] Efficient mixing of quartet‐doublet eigenstates by SOC is however only possible when involving a nearby state with different orbital angular momentum. In metal porphyrins, the lowest two triplet states (*E*_u,*x*_, *E*_u,*y*_; π–π* excitation) are nearly degenerate and are likely to fulfil this role. The energetic difference between the two states involved in the mixing is referred to as Δ and needs to be (significantly) smaller than the thermal energy.

The mixing of ^2^T_1_ and ^4^T_2_ by SO‐ISC is then followed by fast internal conversion from ^4^T_2_ to ^4^T_1_. Through SOC‐mediated mixing, the doublet states acquire some quartet character (and vice versa), which will depend on the energy difference between doublet and quartet states (3*J*_TR_) and will also be spin sublevel specific. This spin‐selectivity leads to spin polarisation of the observed quartet state (^4^T_1_). In the following, back and forth transitions between quartet and doublet states (i.e. thermal repopulation of the trip‐doublet through mixing of ^4^T_1_ with ^2^T_2_) will deplete any initially overpopulated sublevels, causing a thermal equilibration of the populations. The decay to the ground state then primarily occurs from the doublet states, since the transition is spin allowed.

By SO‐ISC the quartet $\left|\pm \frac{1}{2}\right\rangle$ and $\left|\pm \frac{3}{2}\right\rangle$ sublevels are always expected to be equally populated. Therefore, the $\left|+\frac{1}{2}\right\rangle ↔\left|+\frac{3}{2}\right\rangle$ and $\left|-\frac{1}{2}\right\rangle ↔\left|-\frac{3}{2}\right\rangle$ transitions will have equal and opposite polarisations, the so‐called multiplet polarisation. The oppositely polarised multiplet contributions cancel each other in the centre of the spectrum and thus do not contribute to the central quartet feature which results from so‐called net polarisation.

Since net and multiplet polarisation have different properties (spectral positions, orientation dependence, magnetic field dependence, time behaviour),[^18^, ^19^] the time evolution of the polarisation and the ratio of net to multiplet polarisation in the quartet spectra can provide valuable information on the internal dynamics of the system, especially the relative magnitudes of Δ and 3*J*_TR_.

**Figure 7:**
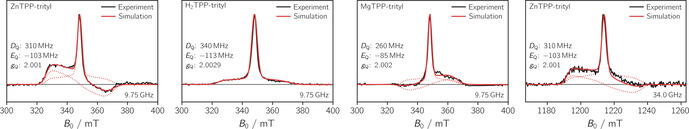
Transient cw EPR spectra of ZnTPP‐trityl, H_2_TPP‐trityl and MgTPP‐trityl (from left to right) at the X‐band (9.75 GHz) and ZnTPP‐trityl (right) at the Q‐band (34.0 GHz) together with the best numeric fit to the experimental data. All spectra were recorded in frozen 2‐methyltetrahydrofuran solution at 80 K at about 1 μs after photoexcitation at 550 nm. The quartet state spectrum was simulated as the sum of net and multiplet components (red dotted lines) with different weights. The simulation parameters are indicated in the figure.

The disappearance of the *a*/*e* multiplet polarisation in less than a few μs in the spectrum of ZnTPP‐trityl (cf. Figure 5) suggests that the doublet‐quartet mixing (equilibration of the quartet populations) is rather fast on the EPR time scale, implying that the energy gaps (3*J*_TR_, Δ) are small compared to the thermal energy (*k*_B_ *T* ≅ 55 cm^−1^ at 80 K and 205 cm^−1^ at room temperature). Further, from a numerical simulation of the data, as shown in Figure 7, the relative ratios of the net to multiplet polarisation, the quartet zero field splitting parameters, *D*_Q_ and *E*_Q_, as well as the *g*_Q_ value of the quartet state can be obtained. The *D*_Q_ value is of particular interest, since it is related to the dipolar interaction between the triplet and radical *D*_TR_ as *D*_Q_=$\frac{1}{3}$ (*D*_T_+*D*_TR_). The obtained *g*_Q_ value of 2.001 is well in line with the predictions for a quartet state from calculations based on the magnetic parameters of the chromophore triplet and stable radical.^[50]^ This and the fact that the spectrum can be fit as a combination of net and multiplet polarisation, using the same magnetic parameters for both contributions, strongly supports our statement that the transient cw EPR spectra observed for ZnTPP‐trityl arise from a single species with quartet multiplicity. An overview of the experimental and calculated *g* and *D* values of all involved spin states is provided in Table S2.^[51]^

Transient cw EPR spectra were also acquired for H_2_TPP‐trityl and MgTPP‐trityl as shown in Figure 7. While the spectral widths are found to be similar in all cases, significant differences in the initial spin polarisation patterns are observed. In particular, it is noted that multiplet polarisation is observed for the quartet states of ZnTPP‐trityl and MgTPP‐trityl, whereas only net polarisation could be detected on the EPR time scale in the case of H_2_TPP‐trityl. The central quartet peak is found to be positive for all investigated compounds, implying a positive sign of the exchange interaction *J*_TR_ between chromophore triplet and radical.^[19]^

Although the formation of quartet states should go hand in hand with excited doublet state formation,[^23^, ^52^, ^53^, ^54^] no doublet signatures could be detected experimentally. Possible reasons for this could either be (i) fast relaxation or (ii) spectral overlap with the quartet feature: Fast relaxation of the doublet excited state (on the EPR time scale) could be favoured since the transition to the ground state doublet is spin‐allowed. Fast relaxation also implies a broad spectral feature, which might be difficult to detect. In addition, since the *g* values of radical and triplet are very similar, also the *g* values of the formed quartet and doublet states will differ only little. Therefore, it is also plausible that the (weaker) signature of the doublet state is buried underneath the quartet spectrum and accounts for minor asymmetries observed in the experimental spectra. In addition, assuming the validity of the model for quartet formation outlined above, the doublet state is not expected to be spin polarised, implying a rather weak signal.

The opposite multiplet polarisation pattern observed for ZnTPP‐trityl and MgTPP‐trityl (*a*/*e* vs. *e*/*a*) might suggest a strong correlation between the quartet state polarisation and the polarisation of the triplet precursor (*aaaeee* vs. *eeeaaa*), i.e. a direct influence of the relative initial triplet state populations and the sign of *D*_T_, in agreement with previous observations.^[55]^ However, an exception is known in the literature, where the polarisation pattern of the quartet state formed in a free‐base porphyrin‐verdazyl system was found to differ from that of the corresponding porphyrin triplet state,^[56]^ implying that the mechanism is likely to be more complex and that other factors, such as the signs of *D*_Q_, *D*_TR_, and *J*_TR_ or the proximity of vibrational states for mixing, also need to be taken into consideration.[^19^, ^22^]

The qualitative similarity of the transient cw EPR spectra recorded for ZnTPP‐trityl at X‐ (9.75 GHz) and Q‐band (34.0 GHz) frequencies (cf. Figure 7) indicates that this system clearly falls within the strong coupling regime. Within this regime, the exchange interaction between triplet and radical, *J*_TR_, has to be larger than any other relevant magnetic interactions in the system, i.e. (i) all hyperfine couplings, the difference in the Zeeman frequencies between triplet and radical, and (iii) the zero‐field splitting in the triplet state. This situation is easily reached in porphyrin‐trityl systems, since the hyperfine couplings in the triplet state are no larger than about 4 MHz,[^43^, ^44^] and the difference in Zeeman frequencies is proportional to the difference in *g* values, which is almost negligible here (cf. Table S2). The strong coupling limit is thus reached as soon as *J*_TR_ exceeds the magnitude of the triplet *D* value (∼0.033 cm^−1^).

When having a closer look at the spectra acquired for ZnTPP‐trityl at the X‐ and Q‐bands, it further appears that the ratio of net vs. multiplet polarisation is slightly increased at the Q‐band (no negative intensities on the high‐field side due to cancellation of the signal with the contribution of the absorptive net polarisation). This is in line with the theoretical prediction that the relative contribution of the net polarisation to the spectra should increase linearly with the field strength and thus supports the validity of the proposed mechanism for quartet state formation (cf. Figure 6).

For all compounds it is observed that |*D*_Q_|∼|3*E*_Q_|, which should imply a comparatively large contribution of the net polarisation.^[19]^ Nonetheless, compared to typical net:multiplet ratios expected based on the above‐mentioned mechanism, the contribution of the net polarisation to the quartet spectra of Figure 7 is surprisingly large. In particular, the complete absence of multiplet polarisation in the spectrum of H_2_TPP‐trityl is intriguing and cannot readily be explained since spin‐selective spin‐orbit induced intersystem crossing from the doublet to the quartet excited state should always lead to the observation of multiplet spin polarisation.[^17^, ^19^, ^48^, ^49^, ^55^]

This apparent inconsistency can be resolved by assuming that doublet‐quartet mixing is fast on the EPR time scale: By the time of detection, a significant part (or all) of the multiplet polarisation might already have decayed. However, to explain the experimental observations, these equilibration dynamics would need to be considerably faster in H_2_TPP‐trityl as compared to ZnTPP‐trityl or MgTPP‐trityl, implying a smaller Δ and/or *J*_TR_ in H_2_TPP‐trityl (cf. Figure 6). Small differences in the molecular geometry or spin delocalisation of the triplet wavefunction could result in marked differences in *J*_TR_. Free base porphyrins are non‐symmetric and non‐planar. The porphyrin core is also likely to be more flexible, resulting in a larger distribution of *J*_TR_. Differences in geometry and/or triplet delocalisation are also suggested by the larger experimental *D*_T_ value of H_2_TPP as compared to ZnTPP and MgTPP. On the other hand, no big differences in terms of delocalisation and *J*_TR_ are expected between ZnTPP and MgTPP, in line with their near identical *D*_T_ values.

Compared to the variations in *J*_TR_, even more significant differences could be imagined between H_2_TPP‐trityl and ZnTPP‐trityl/MgTPP‐trityl regarding the excited state energetics (and therefore Δ): In metal porphyrins, the splitting between Q_x_ and Q_y_ is typically about 30 cm^−1^,[^24^, ^25^] implying that the degeneracy of the ^3^E_u_ states is only slightly lifted and that these two states could well be involved in quartet‐doublet mixing, since the energy gap is reasonably small compared to the thermal energy. In H_2_TPP, the D_4h_ symmetry is broken, leading to a much larger splitting between Q_x_ and Q_y_ of the order of 2800 cm^−1^.^[24]^ The mechanism thus needs to involve a different state in the case of the molecules containing H_2_TPP as the chromophore since any mixing processes involving the second triplet state would clearly be too slow.

## Conclusions

In the present study we investigated the photophysics and magnetic properties of a series of three porphyrin‐trityl compounds, that only differ with respect to the porphyrin central metal. A detailed analysis of the femtosecond transient absorption data recorded at room temperature and in frozen solution enabled us to determine the efficiencies of the individual competing excited state reactions. It was found that excitation energy transfer largely dominates the excited state dynamics with a temperature‐independent time constant of ∼10 ps and an efficiency of ≥95 %, imposing severe limitations on the yield of enhanced intersystem crossing and therefore the efficiency of excited multiplet formation.

Nonetheless, the sensitivity of transient cw EPR was sufficient to detect the formation of quartet states for all three investigated compounds in frozen solution and numerical simulations of the data allowed the characterisation of the relevant magnetic parameters. No significant differences were observed between the spectra recorded at different microwave frequencies, as expected for a system that clearly falls within the strong coupling regime. Differences in the quartet state characteristics between the different compounds were discussed and could be rationalised in the context of previously invoked theoretical models and predictions,[^3^, ^18^, ^19^, ^22^] strongly supporting the validity of the proposed mechanism for this class of compounds. However, in view of the potential applications of such triplet‐radical systems, the low EISC yield observed here is, of course, a substantial drawback. Future studies in this direction should thus focus on improving the design of the molecular system to increase the enhanced intersystem crossing efficiency. To this end, it will be necessary to find out how to suppress excitation energy transfer while keeping the exchange interaction between chromophore triplet and radical in a favourable range for EISC:

In the case of the investigated porphyrin‐trityl compounds, it is likely that the frozen solution samples are composed of molecules with different conformations (i.e. varying dihedral angles between the porphyrin plane and the phenyl spacer, and therefore different *J*_TR_). The EPR signal is thus expected to arise from only a small percentage of molecules in the sample that happen to adopt a conformation favouring EISC over EET. The efficiency of both mechanisms (EET and EISC) depends on the molecular conformation (i.e. orientation of transition dipoles and/or orbital overlap), but if the most favourable conformation for the two excited state deactivation mechanisms were different, one might have a chance to increase the efficiency of EISC by a slight modification of the present molecular design (e.g. by changing the bridge structure: topology, rigidity, extent of conjugation and/or length).

However, it is well possible that a change of the bridge structures is not sufficient to effectively suppress EET. In this case, the excited state energetics of radical and chromophore would need to be modified to eliminate the spectral overlap required for EET, which would most likely imply the choice of a different radical since the UV‐vis absorption of the trityl radical extends over the entire visible range. To safely exclude the possibility of energy transfer, the energies of both the S_1_ and T_1_ states of the chromophore would need to be smaller than the lowest excitation energy of the radical.

## Supporting Information

Synthesis and characterisation of the compounds, UV‐vis absorption spectra, approximation of the Förster rate, description of the experimental setups for fs‐TA and transient EPR, additional fs‐TA data and global kinetic analysis, additional transient EPR data and simulations, as well as DFT calculations of the spin densities.

## Conflict of interest

The authors declare no conflict of interest.

## Supporting information

As a service to our authors and readers, this journal provides supporting information supplied by the authors. Such materials are peer reviewed and may be re‐organized for online delivery, but are not copy‐edited or typeset. Technical support issues arising from supporting information (other than missing files) should be addressed to the authors.

Supplementary
