# Supplementary material for: Excitation Energy Transfer and Exchange‐Mediated Quartet State Formation in Porphyrin‐Trityl Systems
Source: Chemistry. 2020 Dec 1;27(8):2683–91. doi: 10.1002/chem.202002805 (PMC7898503; doi:10.1002/chem.202002805)
Supplement: Supplementary file 1 — Supplementary [file CHEM-27-2683-s001.pdf]

Chemistry–A European Journal

Supporting Information

Excitation Energy Transfer and Exchange-Mediated Quartet State Formation in Porphyrin-Trityl Systems

Oliver Nolden+,[a] Nico Fleck+,[b] Emmaline R. Lorenzo,[c] Michael R. Wasielewski,[c] Olav Schiemann,[b] Peter Gilch,[a] and Sabine Richert*[d]

Table of Contents

1 Synthesis and characterisation of the porphyrin-trityl compounds **S1**

2 UV-vis absorption spectra **S4**

3 Approximation of the Förster energy transfer rate **S4**

4 Additional fs-TA data and global kinetic analysis **S6**

4.1 Room temperature setup and spectra S6

4.2 Low temperature setup and spectra S7

4.3 Global kinetic analysis S9

5 Continuous wave EPR **S11**

6 Additional transient EPR data and simulations **S12**

6.1 EPR sample preparation and setup S12

6.2 Triplet state spectra and simulations S13

6.3 Discussion of the triplet contribution to the transient EPR spectra S14

6.4 Temperature dependence of the transient EPR spectra S15

6.5 Additional quartet spectra and simulations S16

6.6 Discussion of the low signal intensity in transient EPR S16

7 DFT calculations **S17**

List of Figures

S1 ^{1}H-NMR of the porphyrin-trityl compounds S1
S2 MALDI(+)-HRMS of MgTPP-trityl S2
S3 ^{1}H-NMR of MgTPP-trityl S3
S4 UV-vis absorption spectra of porphyrin-trityl compounds S4
S5 UV-vis absorption spectrum of the trityl radical S5
S6 fs-TA data of H_2TPP and H_2TPP-trityl S6
S7 fs-TA data of ZnTPP-trityl in different solvents S7
S8 fs-TA data of trityl and H_2TPP-trityl in frozen solution S8
S9 Decay associated spectra for ZnTPP and H_2TPP S9
S10 Decay associated spectra for the trityl radical S10
S11 Decay associated spectra for ZnTPP-trityl and H_2TPP-trityl S10
S12 Decay associated spectra for trityl, ZnTPP-trityl and H_2TPP-trityl in frozen solution . . . S11
S13 cw EPR spectrum of the trityl radical S12
S14 Simulations of the triplet state EPR spectra of ZnTPP, H_2TPP, and MgTPP S13
S15 TREPR spectra of H_2TPP-trityl and ZnTPP-trityl before and after triplet subtraction . . . S15
S16 Transient cw EPR spectra of H_2TPP-trityl and ZnTPP-trityl at different temperatures . . . S15
S17 Schematic representation of the energy levels of the quartet state S16
S18 Comparison of the Q-band transient cw EPR spectra of H_2TPP-trityl and ZnTPP-trityl . . S16
S19 Comparison of the pulse EPR spectra of H_2TPP-trityl with and without photoexcitation . . S17
S20 Visualisation of the spin densities predicted by DFT calculations S17

1 Synthesis and characterisation of the porphyrin-trityl compounds

H_2TPP-trityl, ZnTPP-trityl and the diamagnetic precursor molecule H_2TPP-trityl-OH were synthesised according to the procedures described in an earlier publication [1]. MgTPP-trityl was obtained by treatment of H_2TPP-trityl with $MgBr_2(Et_2O)$ in dichloromethane [2].

Out of these four compounds, MgTPP-trityl was especially prone to oxidation during the purification process. The oxidation transforms the radical center into the corresponding diamagnetic triphenylmethanol moiety (H_2TPP-trityl-OH). As shown in Figure S1, the H_{phenyl} signals of the trityl core are broadened beyond visibility due to paramagnetic relaxation enhancement induced by the trityl radical (highlighted region). In contrast, these signals can be observed for the diamagnetic impurities, enabling the quantification of the radical content. Using this approach, H_2TPP-trityl and ZnTPP-trityl show no clear indication of diamagnetic impurities, whereas MgTPP-trityl exhibits a porphyrin:radical ratio of 1:0.75, determined by integration of the ^{1}H-NMR spectrum.

It should be noted that this method is likely not sensitive enough to detect an impurity content lower than a few percent, so small amounts of a diamagnetic impurity cannot be completely excluded even for H_2TPP-trityl and ZnTPP-trityl. In addition, it needs to be added that neither MALDI(+) nor ESI(+) mass spectrometry allow a reliable quantification in the present case, since signals belonging to sulfoxide impurities (M+16) can hardly be distinguished from trityl alcohol (M+17) impurities. Moreover, the presence of free-base porphyrin could also be probed by ^{1}H-NMR through observation of the characteristic signal at approximately −2.8 ppm. As shown in Figure S1, neither the zinc nor the magnesium complex contained any significant amount of free-base porphyrin.

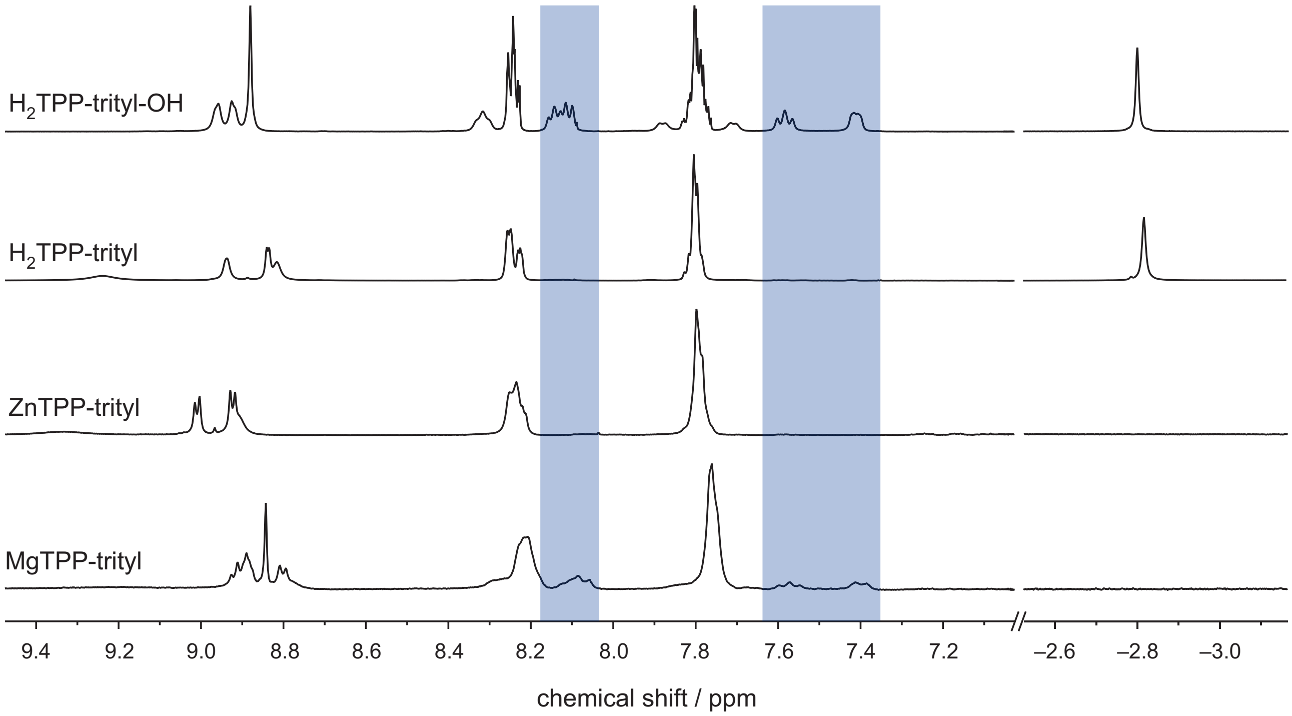

Figure S1: ^{1}H-NMR (700 MHz, CD_2Cl_2, 298 K) of H_2TPP-trityl-OH, H_2TPP-trityl, ZnTPP-trityl, and MgTPP-trityl. The region affected by paramagnetic broadening is highlighted.

Synthesis of MgTPP-trityl. H_2TPP-trityl (15 mg, 8.6 μmol) was dissolved in 4 mL dry dichloromethane and triethyl amine (69.1 mg, 95 μL, 640 μmol, 80 eq.) and magnesium bromide etherate (88 mg, 342 μmol, 40 eq.) was added. The reaction mixture was stirred at room temperature under argon for 45 minutes, diluted with 10 mL dichloromethane, washed with 5 % $NaHCO_3$ (2×10 mL), and then dried over $MgSO_4$.

The solvents were evaporated under reduced pressure and the residue was purified by column chromatography on silica eluting with cyclohexane/methanol 90:10 (v/v). This provided the title compound as a purple solid in a yield of 12 mg (78 %, 6.8 μmol).

^{1}H-NMR (700 MHz, CD_2Cl_2, 298 K, δ in ppm): 8.95–8.75 (m, 8H, H_{pyrrol}), 8.24–8.18 (m, 6H, $H_{phenyl, por}$), 7.78–7.72 (m, 9H, $H_{phenyl, por}$).

The following signals occur additionally for the corresponding triphenylmethanol contained as an impurity: 8.14–8.04 (m, 4H), 7.62–7.53 (m, 2H), 7.44–7.36 (m, 2H), 3.90 (s, 3H), 3.89 (s, 3H), 2.03 (s, 3H), 1.92 (s, 6H), 1.91 (s, 6H), 1.83–1.78 (s, 15 H), 1.74 (s, 3H), 1.71 (s, 3H).

Since the baseline in the alkyl region is distorted, probably due to an underlying signal of the 36 methyl hydrogen atoms of the thioketal moiety, the porphyrin:radical ratio is determined by integration in the aromatic region.

HRMS (MALDI+, DCTB, m/z): 1771.242 (calcd. for $C_{97}H_{77}N_4O_4S_{12}Mg$: 1771.245).

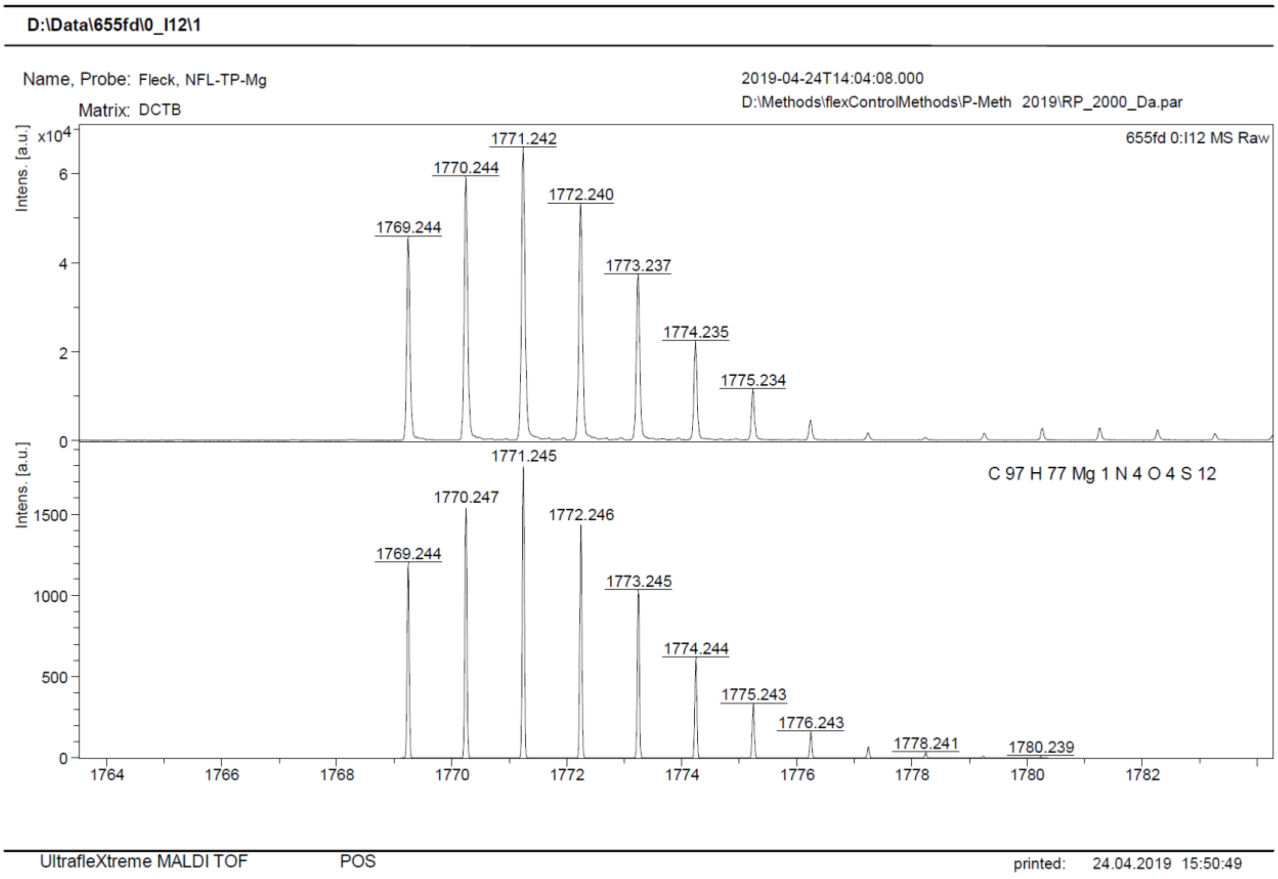

Figure S2: MALDI(+)-HRMS of MgTPP-trityl.

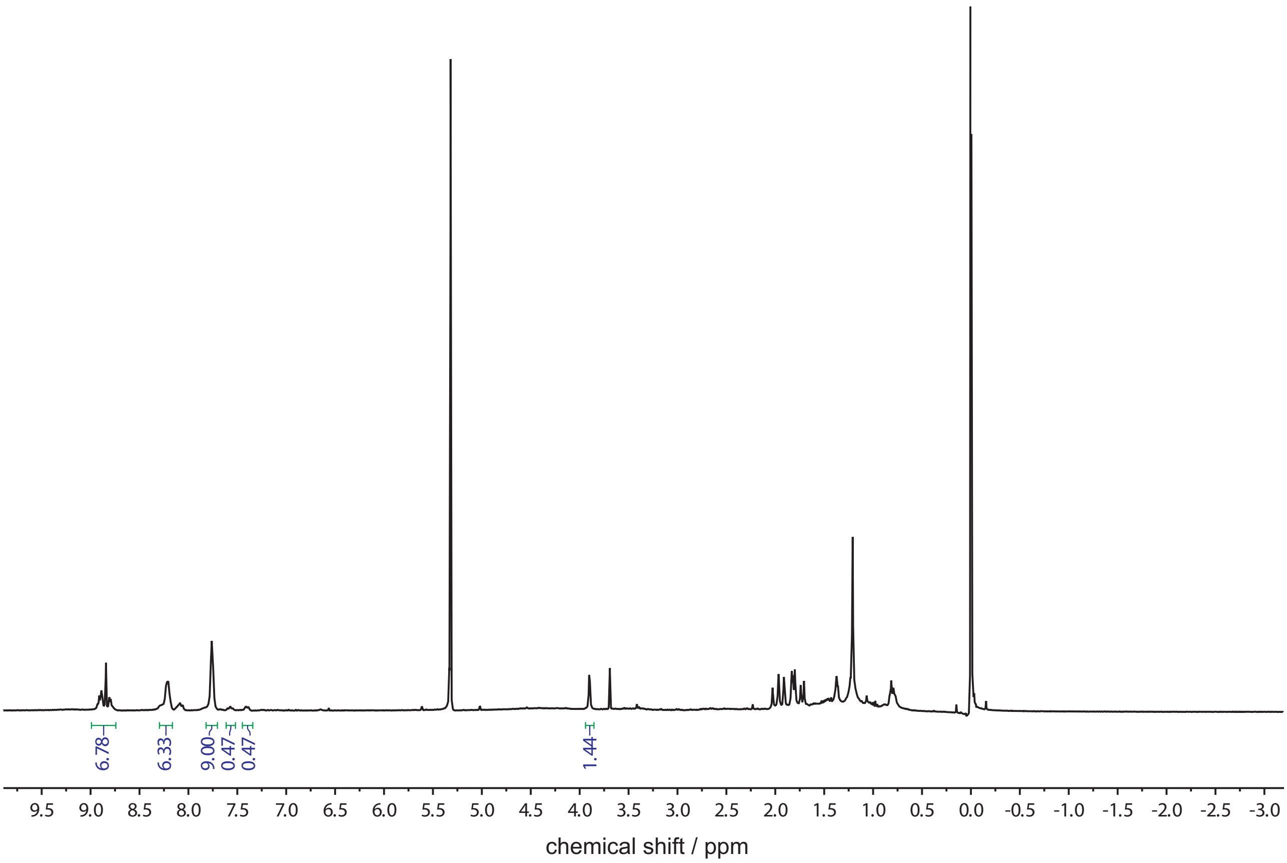

Figure S3: ^{1}H-NMR (700 MHz, CD_2Cl_2, 298 K) of MgTPP-trityl.

2 UV-vis absorption spectra

The UV-vis spectra of the three investigated porphyrin building blocks, ZnTPP, H_2TPP and MgTPP, recorded in toluene solution at room temperature are shown in Figure S4. As it is typical for this class of chromophores, the most intense absorption peak in the visible range is observed around 420 nm (Soret band), while the absorption peaks of the much less intense Q-bands cover the region from about 500 to 660 nm.

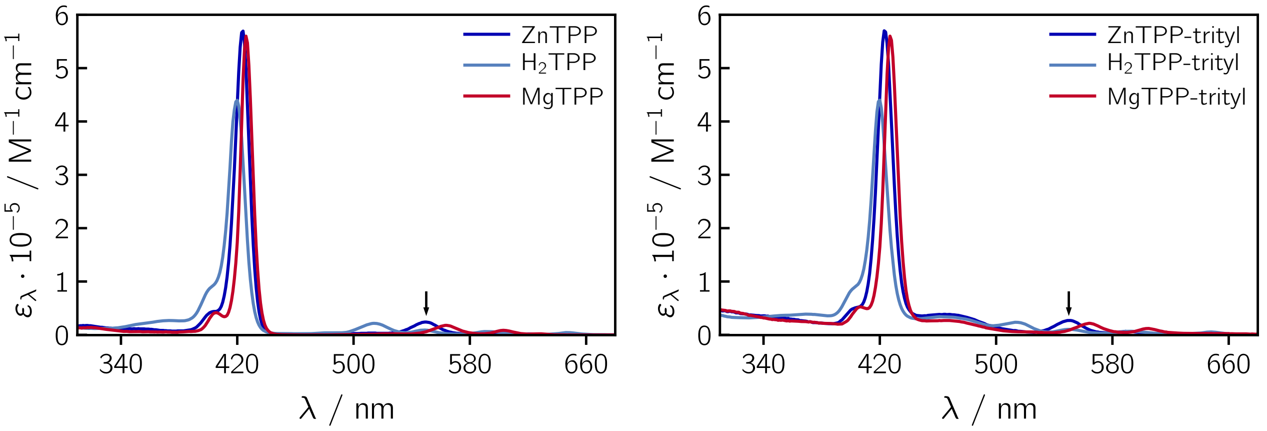

Figure S4: UV-vis absorption spectra of the investigated tetraphenyl-porphyrins (*left*) and the corresponding porphyrin-trityl compounds (*right*) scaled to their known molar absorption coefficients. It is assumed that the molar absorption coefficient of the porphyrin is not influenced by addition of the trityl moiety. The excitation wavelength of 550 nm, chosen for most experiments, is indicated by a black arrow.

The molar absorption coefficients of the three porphyrins at the Soret band maximum amount to ε(ZnTPP, 423 nm) $= 5.74 \cdot 10^5\,M^{-1}\,cm^{-1}$, ε(H_2TPP, 420 nm) $= 4.43 \cdot 10^5\,M^{-1}\,cm^{-1}$ and ε(MgTPP, 427 nm) $= 5.62 \cdot 10^5\,M^{-1}\,cm^{-1}$ [3]. In the optical experiments, photoexcitation was carried out at 550 nm.

From the characterisation of the porphyrin-trityl compounds it is known that, in a few percent of the sample, the trityl is present in its alcohol rather than its radical form. From the UV-vis and fs-TA spectra of the compounds it can be estimated that this percentage of diamagnetic impurity amounts to roughly $\sim$0–3 % in the case of ZnTPP-trityl and H_2TPP-trityl and to roughly 30 % in the case of MgTPP-trityl (for reasons detailed in the synthesis section above). In the transient EPR experiments, these molecules, where the trityl radical has been deactivated, will show up as a triplet 'impurity' and contribute to the background signal. Since the triplet signal from porphyrins is quite strong (triplet yields of $>$80 % [4, 5] and strong spin polarisation), this background can be quite substantial compared to the intensity of the quartet signal.

3 Approximation of the Förster energy transfer rate

The molar absorption coefficient of the trityl radical was estimated from UV-vis data of ZnTPP and ZnTPP-trityl (cf. Figure S4), taking into account the known molar absorption coefficient of ZnTPP at the Soret band maximum of $5.7 \cdot 10^5\,M^{-1}cm^{-1}$ [3, 6]. It was assumed that the molar absorption coefficient at the intensity maximum of the porphyrin Soret band is the same for ZnTPP and ZnTPP-trityl and that the spectrum of ZnTPP-trityl (at least at 460 nm) is equal to the sum of the spectra of ZnTPP and trityl radical with a molar ratio of 1:1. The value for the fluorescence quantum yield of ZnTPP was taken from reference [7] and the center-to-center distance r_{DA} from a DFT model of the ZnTPP-trityl stucture.

Table S1: Overview of the results from an approximate calculation of the Förster radius, FRET rates, and efficiencies for ZnTPP-trityl. The following parameters were used: $r_{DA} = 1.3\,\mathrm{nm}$, $n = 1.496$ (toluene), $\tau^{D}_{F,0} = 2.6\,\mathrm{ns}$, $\Phi^{D}_{F,0} = 0.04$, $\varepsilon^{A}(460\,\mathrm{nm}) = 3.8 \cdot 10^{4}\,\mathrm{M^{-1}cm^{-1}}$.

κ^2	R_0 / nm	τ_{FRET} / ps	Φ_{FRET} / %
$\frac{2}{3}$ (random)	2.42	63	97.6
1 (parallel)	2.59	42	98.4
4 (collinear)	3.26	10	99.6

The Förster radius R_0 (obtained in nm) can be calculated according to [8]

$$R_0^6 = 8.785 \cdot 10^{-11}\,\frac{\Phi^{D}_{F,0}\,\kappa^2}{n^4} \int I^{D}_{F}(\lambda)\,\varepsilon^{A}(\lambda)\,\lambda^4\,\mathrm{d}\lambda \tag{S1}$$

with

$$\int I^{D}_{F}(\lambda)\,\mathrm{d}\lambda = 1 \tag{S2}$$

where $\Phi^{D}_{F,0}$ and I^{D}_{F} are the fluorescence quantum yield and fluorescence intensity of the donor, ε^{A} is the molar absorption coefficient (in $\mathrm{M^{-1}cm^{-1}}$) of the acceptor and n the refractive index of the medium. The orientation factor κ^2 accounts for the relative orientation of the two transition dipole moment vectors (emission of donor and absorption of acceptor) with respect to the axis connecting the FRET pair.

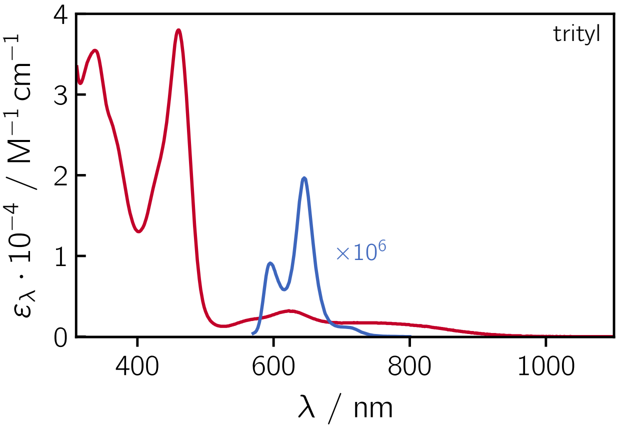

Figure S5: UV-vis absorption spectrum of the trityl radical. The fluorescence spectrum of ZnTPP is superimposed (in blue) for an illustration of the spectral overlap.

The energy transfer rate and FRET efficiency are then given as

$$\tau^{-1}_{FRET} = k_{FRET} = \frac{1}{\tau^{D}_{F,0}} \left(\frac{R_0}{r_{DA}}\right)^6 \tag{S3}$$

and

$$\Phi_{FRET} = 1 - \frac{\tau_{FRET}}{\tau^{D}_{F,0}} \tag{S4}$$

where $\tau^{D}_{F,0}$ is the fluorescence lifetime of the donor in the absence of any quenchers and r_{DA} is the center-to-

center distance (point dipole) between donor and acceptor. The results obtained for ZnTPP-trityl assuming different values for κ^2 are summarised in Table S1. The spectral overlap of the trityl absorption and ZnTPP fluorescence spectra is illustrated in Figure S5.

4 Additional fs-TA data and global kinetic analysis

4.1 Room temperature setup and spectra

For the room temperature femtosecond TA experiments the samples were prepared in either toluene or 2-methyl-THF solutions. UV-vis spectra were taken before and after the measurements to verify the sample absorbances and confirm the absence of sample degradation during the measurement. Care was taken that the absorbance in the Soret-band region did not exceed a value of about 1.5, to avoid optical saturation of the ground state bleach (GSB) and thus ensure accurate intensity readings in this region. As a consequence, the absorbances at the excitation wavelengths of either 400 nm or 550 nm were fairly low (0.02−0.1).

The setup used for the room temperature measurements was described in detail elsewhere [9–12]. In brief, a Ti:Sapphire amplified laser system (Coherent Libra) with a repetition rate of 1 kHz, a pulse duration of 100 fs and a wavelength of 800 nm was used as the pulse source. Part of its output was used to pump a TOPAS-White non-collinear optical parametric amplifier tuned to deliver pulses peaking at 550 nm. For probing (330−740 nm), a supercontinuum was generated in a CaF_2 plate. The pump beam diameter at the sample was 160 μm (FWHM), while the diameter of the probe beam amounted to 100 μm. The relative polarisation of pump and probe beams was set to the magic angle and the IRF was about 180 fs.

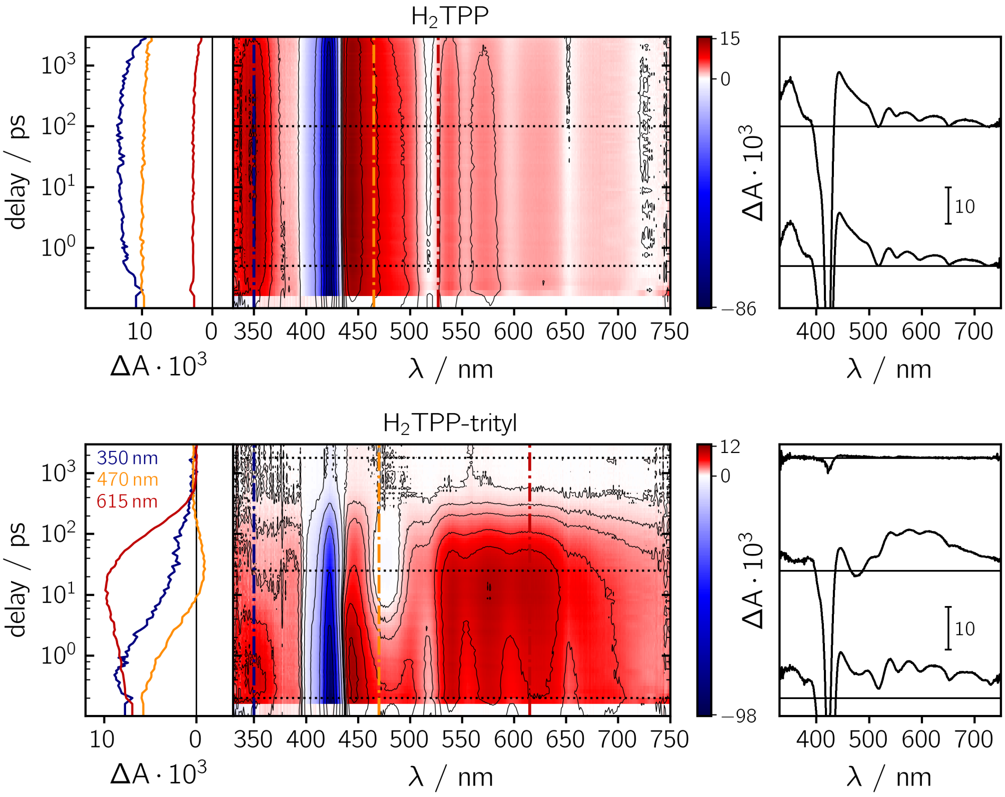

Figure S6: Femtosecond transient absorption data for H_2TPP (*top*) and H_2TPP-trityl (*bottom*) recorded in toluene solution at room temperature after photoexcitation at 550 nm.

During the experiments, the sample solutions (∼ 2 mL) were flown continuously. The optical path length of the cuvette was 0.5 mm. The data were acquired with 20 averages per time point and four scans using

a pump power of about 1 mW. A total of 139 spectra were collected, with 50 linear time steps between −1 and 1 ps, followed by logarithmic time steps up to roughly 4 ns. The chopper in the probe beam path was set to a frequency of 500 Hz, while that in the pump path was set to 250 Hz. For every time point, four different sets of data were collected: (i) only the white light (probe) reaches the sample, (ii) pump and probe both blocked, (iii) pump and probe both reach the sample, (iv) only the pump reaches the sample. Signals (i) and (iii) are used for the calculation of ΔA, while signals (ii) and (iv) are needed to account for offsets and pump light scattering. The chirp of the white light was measured in a separate (OKE) experiment and accounted for in the processing of the TA spectra. In addition, solvent spectra were recorded separately under identical conditions as the samples and subtracted from the sample data following the procedure detailed in reference [13].

The TA data were analysed using home-written MATLAB routines. After subtraction of the solvent background, the data were chirp-corrected by interpolation in the time domain using a function of the form $f(x) = p_1 + \frac{p_2}{x^2} + \frac{p_3}{x^4}$ with parameters p_n determined by analysis of the corresponding OKE experiment (i.e. fit of $f(x)$ to the OKE data as a function of wavelength).

The femtosecond TA experiments at room temperature were also carried out at an excitation wavelength of 400 nm and using a solvent with a significantly higher dielectric constant (2-methyltetrahydrofuran vs toluene). No significant differences in either the spectral signatures or kinetics were observed in these two cases as shown below.

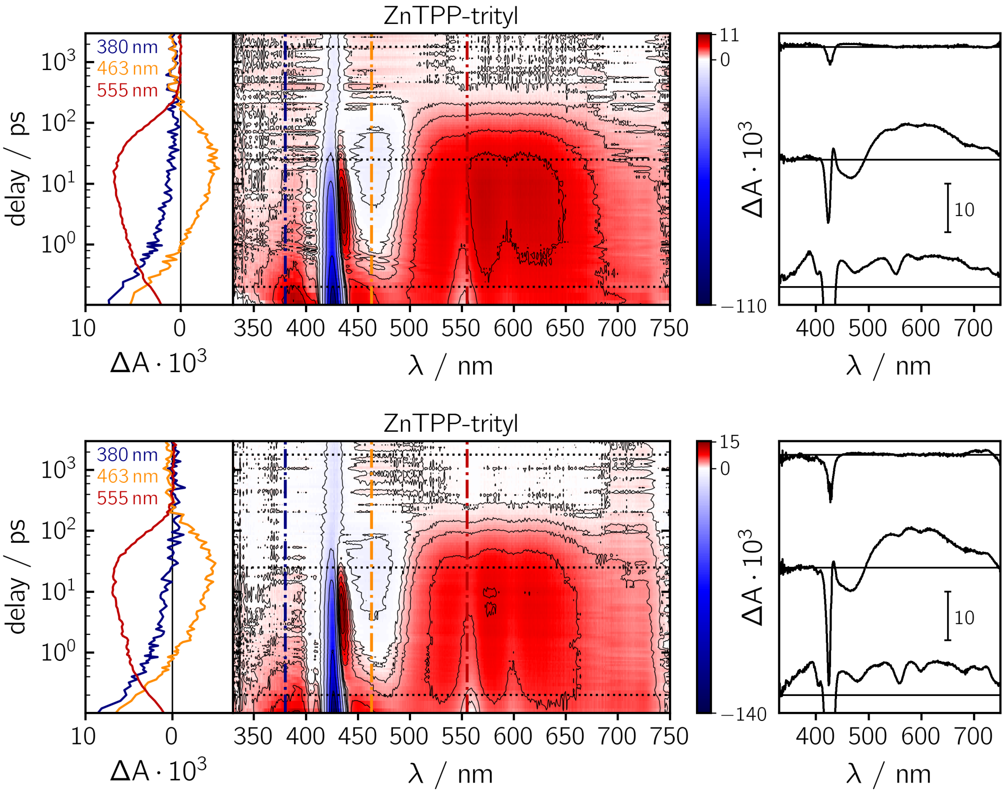

Figure S7: Femtosecond transient absorption data for ZnTPP-trityl recorded in toluene (*top*) and 2-methyltetrahydrofuran (*bottom*) solution at room temperature after photoexcitation at 400 nm.

4.2 Low temperature setup and spectra

To verify whether the efficiencies of the different excited state reactions differ under the conditions applied during the EPR measurements, femtosecond TA experiments were also performed in frozen 2-methyl-THF

solution at 85 K using an optical cryostat. The solvent, 2-methyl-THF, was distilled, deoxygenated and stored in the glove box where the sample solutions were prepared. The sample cell, composed of two quartz windows separated by a teflon spacer (~2 mm), was filled with the solution and assembled under oxygen exclusion in the glove box. The sample concentration was adjusted to yield an OD of about 0.3−0.6 at the excitation wavelength in the sample cuvette.

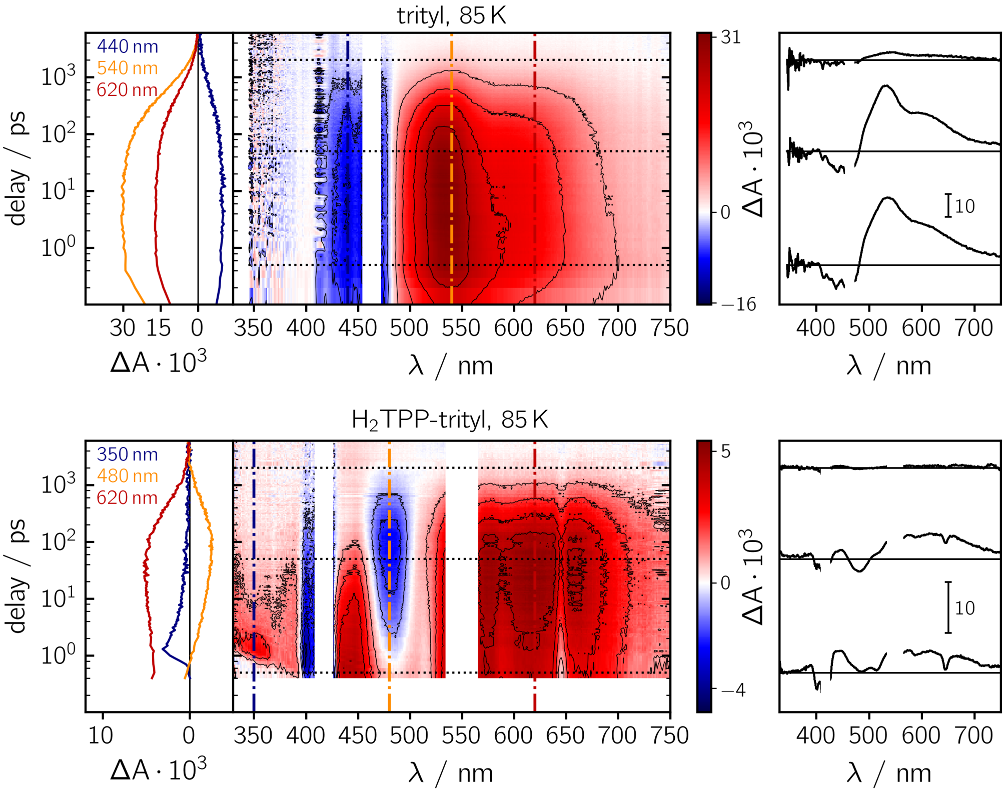

Figure S8: Femtosecond transient absorption data for the trityl radical (*top*) and H_2TPP-trityl (*bottom*) recorded in frozen 2-methyl-THF solution at 85 K after photoexcitation at 414 nm and 550 nm, respectively. The wavelength ranges dominated by artefacts from either excitation light scattering or optical saturation were cut out from the spectra.

The transient absorption measurements were performed with pulses of about 90 fs duration (centred at 827 nm) provided by an amplified Ti:sapphire system (Spitfire, Spectra Physics) at a repetition rate of 1 kHz. The fundamental beam at 827 nm is split into two parts: A small fraction is used for probe white light generation, the other part was guided through an optical parametric amplifier for excitation at 550 nm or frequency doubled for excitation at 414 nm. A commercially available Helios spectrometer (Ultrafast Systems) was used for data acquisition in combination with a slightly modified optical layout. The probe beam is delayed on an optical stage prior to white light generation in a sapphire plate and is then split in a signal and reference beam for signal detection and compensation of intensity fluctuations, respectively. To obtain the transient absorption difference signal, ΔA, the absorption was recorded in the absence and presence of the pump beam using an optical chopper in the pump beam path.

Several scans needed to be averaged until an acceptable signal-to-noise ratio could be obtained. Between individual scans, the cryostat was laterally displaced, whenever necessary, to minimise the effects of sample degradation on the acquired spectra. All TA spectra were acquired using an excitation energy of $\sim 1\,\mu$J. In order to obtain an appreciable signal, a relatively high sample OD of about 0.3−0.6 at the excitation wavelength was required, resulting in optical saturation in certain regions of the spectrum around the intensity maxima of the UV-vis spectra. In addition, artifacts from pump light scatter at the excitation wavelength

could not be avoided when using the cryostat. The wavelength ranges dominated by artefacts from either excitation light scattering or optical saturation were cut out from the spectra. The data were analysed in a similar way as described above for the room temperature experiments.

4.3 Global kinetic analysis

To get a better estimate of the time constants of the excited state reaction processes, a model-free global kinetic analysis of the recorded fs-TA data was carried out [14]. In the case of the porphyrin chromophores and porphyrin-trityl compounds it was found that three and four time constants, respectively, were required in order to accurately reproduce the data recorded at room temperature, while only two time constants were needed for a satisfactory simulation of the trityl radical spectra.

The decay associated spectra (DAS) obtained for ZnTPP and H_2TPP are shown in Figure S9. In the analysis, simultaneous mono-exponential decays are assumed. The DAS are then obtained by plotting the contributions, i.e. amplitudes, of the individual time constants as a function of wavelength.

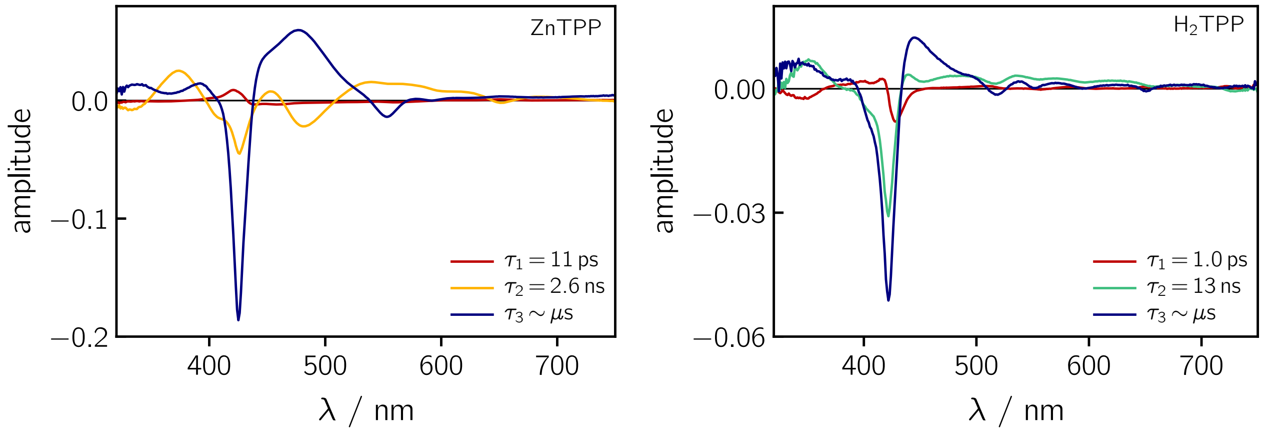

Figure S9: Decay associated spectra and associated time constants obtained from a model-free global kinetic analysis of the femtosecond TA data for ZnTPP (*left*) and H_2TPP (*right*). The lifetimes of the first excited singlet states of ZnTPP and H_2TPP are known from the literature and were kept fixed during the optimisation procedure.

The fs-TA spectra of the porphyrin chromophores, ZnTPP and H_2TPP, are well known and have been extensively analysed in the literature [15–17]. In general, after photoexcitation of the porphyrin, the first excited singlet state decays with a time constant of about 13 ns or 2.6 ns for H_2TPP and ZnTPP, respectively, to form the porphyrin triplet state in high yield [4, 5, 7]. The latter then lives for about 1 μs in solution at room temperature [7]. The signatures of the porphyrin excited singlet and triplet states are very similar in both cases and may therefore be hard to distinguish.

The first time constants in Figure S9 can be attributed to vibrational relaxation processes occurring in the first excited singlet state of the chromophores, while the second and third time constants represent the decay of the porphyrin singlet and triplet states, respectively.

Figure S10 shows the kinetic analysis of the room temperature fs-TA spectra of the trityl radical. The experimental data could be reproduced with only two time constants, attributed to (i) relaxation within S_1 and (ii) the excited state decay of the trityl radical.

The kinetic analysis of the fs-TA spectra of ZnTPP-trityl and H_2TPP-trityl is shown in Figure S11. Of the four time constants needed to satisfactorily reproduce the experimental data, three were left to vary freely during the analysis, while the fourth time constant was fixed to the known decay constant of the excited triplet state of the porphyrins. This forth time constant accounts for the residual porphyrin signal left after

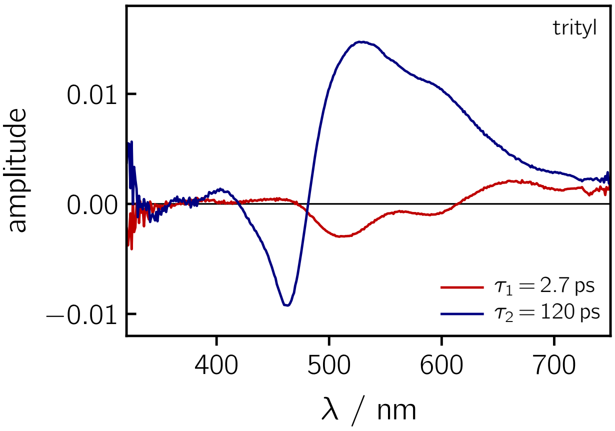

Figure S10: Decay associated spectra and associated time constants obtained from a model-free global kinetic analysis of the femtosecond TA data of the trityl radical.

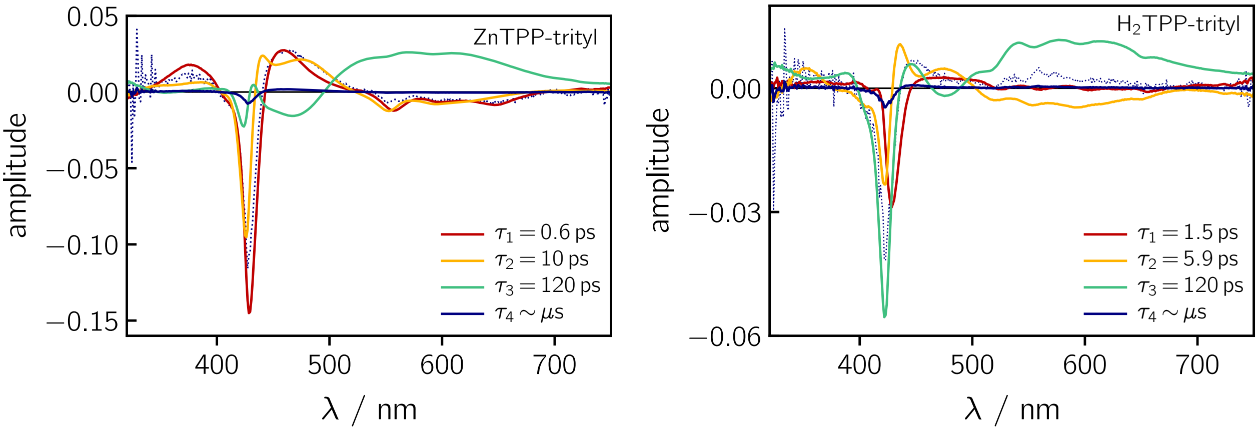

Figure S11: Decay associated spectra and associated time constants obtained from a model-free global kinetic analysis of the femtosecond TA data for ZnTPP-trityl (*left*) and H_2TPP-trityl (*right*). The lifetimes of the first excited singlet states of ZnTPP and H_2TPP are known from the literature and were kept fixed during the optimisation procedure. The blue dotted line represents a scaled version of the component in blue.

complete decay of the trityl radical excited state features (>500 ps). The remaining three time constants can be attributed to the following processes: (i) vibrational relaxation in the first excited singlet state of the porphyrin, (ii) energy transfer from the porphyrin to the trityl, i.e. decay of the porphyrin excited singlet state and simultaneous built-up of the trityl radical features, (iii) decay/lifetime of the trityl radical excited state absorption. In both cases, the energy transfer from the porphyrin to the trityl radical occurs in roughly 10 ps at room temperature.

The analysis of the room temperature fs-TA spectra of ZnTPP-trityl and H_2TPP-trityl was performed twice, either (i) with the third time constant fixed to the determined decay time of the trityl radical species (i.e. 120 ps) or (ii) leaving the third time constant to vary freely. Almost identical decay associated spectra were obtained in both cases and also the variations in the decay times are very minor. For reference, the following τ-values were obtained when leaving the third time constant to vary freely: ZnTPP-trityl – (i) 0.6 ps, (ii) 11 ps, (iii) 94 ps; H_2TPP-trityl – (i) 1.3 ps, (ii) 6.4 ps, (iii) 148 ps.

Although the quality of the data is not perfect, a kinetic analysis was also performed for the fs-TA data recorded in frozen solution. The DAS obtained for the trityl radical, for ZnTPP-trityl and for H_2TPP-trityl in frozen 2-methyl-THF at 85 K are shown in Figure S12. No significant amount of residual porphyrin signal could be detected after complete decay of the trityl excited state absorption, so only three time constants

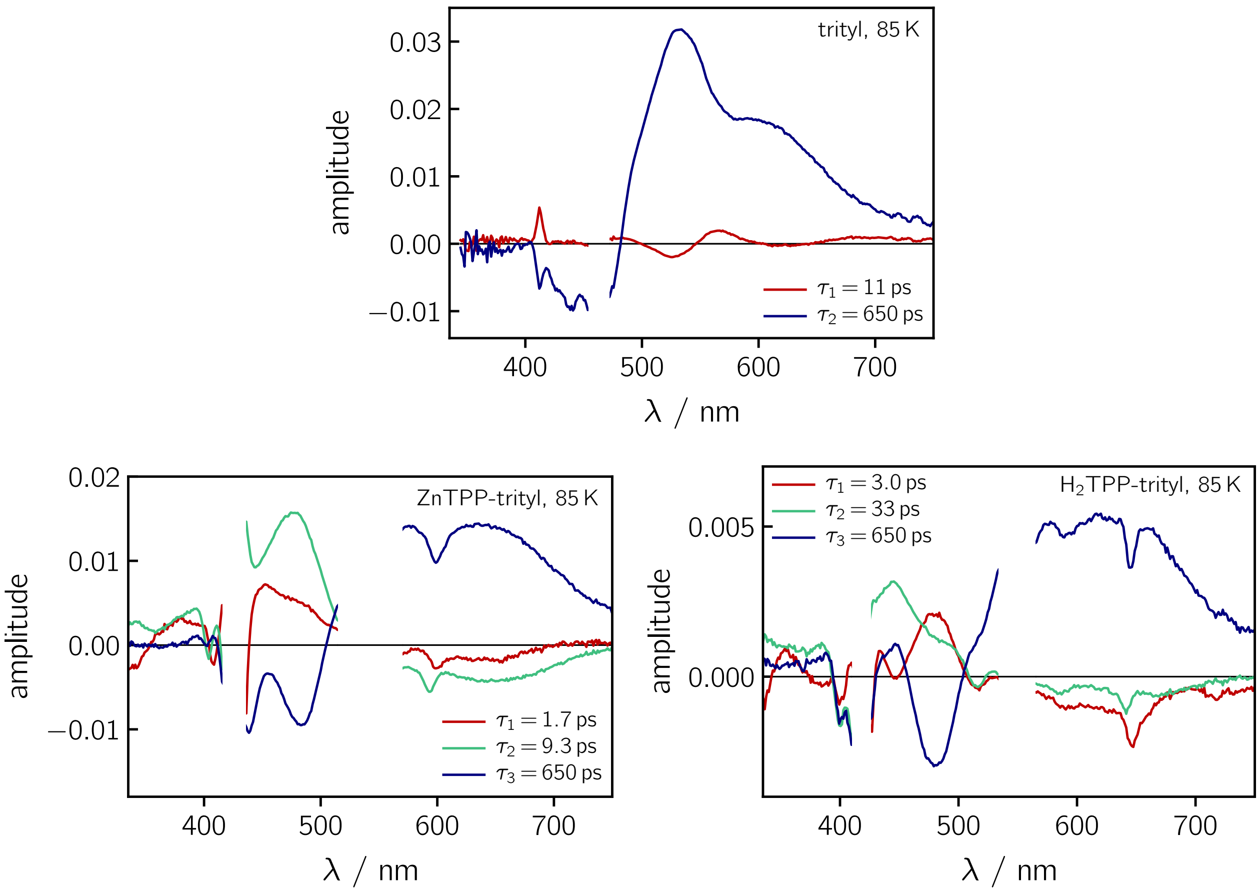

Figure S12: Decay associated spectra and associated time constants obtained from a model-free global kinetic analysis of the femtosecond TA data for trityl (*top*), ZnTPP-trityl (*bottom, left*) and H_2TPP-trityl (*bottom, right*) recorded in frozen 2-methyl-THF solution at 85 K after photoexcitation at 414 nm (*top*) or 550 nm (*bottom*).

were necessary to satisfactorily reproduce the data. It can be seen that the overall kinetics are somewhat slower in frozen solution, but the general trends and spectral features observed at room temperature are conserved: The trityl radical excited state lifetime is increased from about 120 ps to 650 ps. However, the deactivation of the excited porphyrin species in the presence of the stable radical is still almost equally fast (∼10 ps). Thus, even at 85 K, energy transfer from the porphyrin to the trityl radical seems to dominate the excited state dynamics.

Also for this set of data, the analysis of the fs-TA spectra of ZnTPP-trityl and H_2TPP-trityl was performed twice, either with the third time constant fixed to the determined decay time of the trityl radical species (i.e. 650 ps) or leaving the third time constant to vary freely. As before, almost identical decay associated spectra were obtained in both cases. The variations in the decay times were very minor for ZnTPP-trityl (τ-values of (i) 1.6 ps, (ii) 10 ps, (iii) 560 ps); identical time constants were obtained for H_2TPP-trityl.

5 Continuous wave EPR

To characterise the g-value of the trityl radical, a continuous wave EPR spectrum of H_2TPP-trityl in toluene was recorded in the dark at room temperature. The measurement was carried out at the X-band (9.75 GHz) using a modulation amplitude of 0.1 G and a microwave power of ∼ 0.13 mW. The recorded, background-corrected spectrum was frequency-corrected to 9.75 GHz and field-corrected using a carbon fibre standard [18]. A g-value of 2.0028 was obtained for the trityl radical by simulation of the spectrum using EasySpin

[19] as shown below.

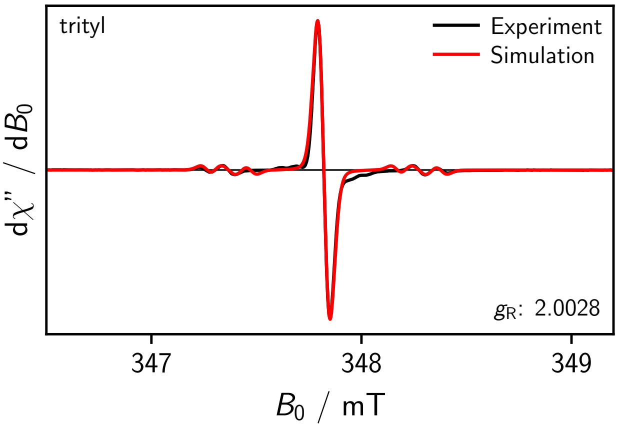

Figure S13: Room temperature continuous wave EPR spectrum of H_2TPP-trityl in toluene recorded in the dark at the X-band (9.75 GHz) using a modulation amplitude of 0.1 G and a microwave power of about 0.13 mW. The g-value was extracted from the numerical simulation of the spectrum superimposed in red.

6 Additional transient EPR data and simulations

6.1 EPR sample preparation and setup

For all transient cw EPR measurements, the samples were prepared with an absorbance of roughly 0.3 at the excitation wavelength of 550 nm, measured in a 2 mm cuvette. For measurements at the X-band, the samples in either toluene or 2-methyl-THF were then transferred into quartz EPR tubes with an outer diameter of 3.8 mm (inner diameter ~3 mm). The solutions were rapidly frozen in liquid nitrogen before insertion into the EPR resonator for the measurements.

Transient EPR measurements were carried out on a Bruker ELEXSYS E580 spectrometer operated at the X-band (9.75 GHz) and equipped with a Bruker EN 4118X-MD4 resonator. During the measurement, the sample was kept at a constant temperature (80 K, unless stated otherwise) using an Oxford Instruments nitrogen gas-flow cryostat (CF 935). The samples were excited through the top of the sample holder with depolarised light at 550 nm using an excitation energy of 1 mJ at a repetition rate of 20 Hz (pulse duration ~5 ns).

The spectra were acquired in direct detection mode with a videoamplifier bandwidth of 20 MHz using the built-in transient recorder (DC-AFC) and a microwave power of 1.5 mW (20 dB). Any positive signal thus corresponds to an absorptive transition (*a*) and any negative signal to an emissive (*e*) one. The time resolution of the experiment is limited by the resonator bandwidth to about 200 ns (~5 MHz). For samples giving only very weak signals, a possibility to enhance the signal-to-noise ratio of the data is to work with the transient recorder in AC-AFC mode and to feed the transient signal through a low-noise voltage preamplifier (Stanford Research Systems SR 560) before it enters the detection circuitry. This configuration was typically used for the acquisition of the data of the porphyrin-trityl compounds, using a bandpass filter of 3 kHz−1 MHz. The time resolution of the spectra is thus somewhat compromised for a better resolution of the spectral shape. Typically, for every magnetic field value, a time trace with 4096 points was recorded using a time base of 4 ns. After data acquisition, the 2D spectra were baseline-corrected in both dimensions using a home-written MATLAB routine.

For the transient cw EPR measurements at Q-band frequencies (34.0 GHz) a Bruker EN 5107D2 resonator was used and the samples were prepared in quartz tubes with an outer diameter of 1.6 mm (inner diameter

of ~1 mm). The samples were again excited through the top of the sample holder using an excitation energy of only ~0.5 mJ. All other experimental parameters were kept the same.

6.2 Triplet state spectra and simulations

Transient cw EPR spectra of the triplet states of H_2TPP, ZnTPP and MgTPP recorded in frozen toluene at 80 K are shown in Figure S14. The shape of the spectra of these compounds was found not to change significantly over the course of the triplet state lifetime. The spectra shown in the figure were averaged over a time window from 0.2 μs to 1 μs after laser excitation. It can be seen that, due to different intersystem crossing mechanisms, the triplet states of H_2TPP and ZnTPP show an opposite spin polarisation [20]. While the spin polarisation pattern in H_2TPP (from low to high field) is *eeeaaa*, an *aaaeee* polarisation pattern is obtained for ZnTPP. The triplet state D-value of both compounds is known to be positive [21], therefore the observed spin polarisation corresponds to an overpopulation of the in-plane triplet sublevels (X, Y) in H_2TPP and a predominant population of the out-of-plane triplet sublevel (Z) in ZnTPP. Also the triplet state of MgTPP shows an opposite spin polarisation (*eeeaaa*) as compared to ZnTPP. To determine accurate g-values as well as the zero-field splitting D-values and relative populations of the excited multiplet states, numerical simulations of the experimental spectra were performed using the MATLAB package EasySpin in combination with home-written fitting routines. The simulation results and parameters can be found in Figure S14.

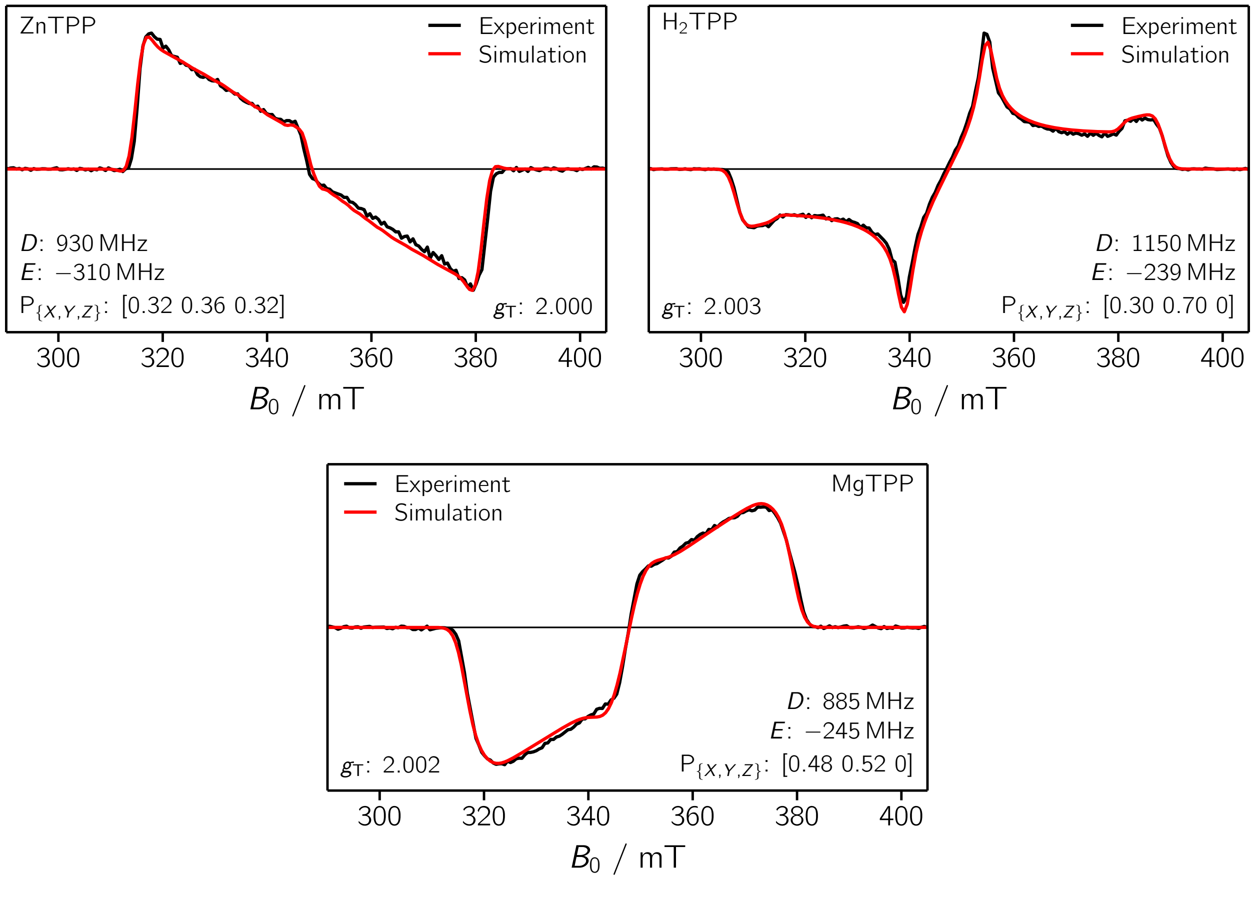

Figure S14: Triplet state transient cw EPR spectra of ZnTPP (*left, top*) and H_2TPP (*right, top*) and MgTPP (*bottom*) recorded about 1 μs after photoexcitation at the X-band (9.75 GHz) in frozen 2-methyl-THF solution at 80 K together with the best numeric fit to the experimental data. The simulation parameters are indicated in the figure.

Apart from differences in the spin polarisation pattern, it is noted that the triplet state D-value, reflected in the width of the triplet state spectrum, is significantly smaller in ZnTPP as compared to H_2TPP, potentially pointing either towards an increased delocalisation of the triplet state wavefunction in ZnTPP or a different excited state geometry (due to the fact that the porphyrin core is no longer planar in H_2TPP).

In theory, spin-orbit coupling contributions to the ZFS parameter D could be expected in ZnTPP, but previous experiments on Zn-porphyrin triplet states suggest that this is not the case [22]. In addition, in a series of linear Zn-porphyrin oligomers, all experimental observations and trends in D could be consistently explained and reproduced by quantum chemical calculations, taking only the spin-spin contribution to the ZFS into account [20, 23]. Also the fact that the experimental D-value (i.e. spectral width) is almost identical for MgTPP and ZnTPP suggests that only spin-spin contributions to D need to be taken into account.

An overview of the magnetic parameters obtained as a result from the spectral simulations of the triplet and quartet state spectra (cf. also main text) is given in Table S2.

Table S2: Overview of the g- and D-values of the different multiplet states present before and after photoexcitation of H_2TPP-trityl, ZnTPP-trityl and MgTPP-trityl. The g-values of the triplet and radical as well as the D-values of the triplet and quartet state were determined from the experimental data, while the expected g-values of the excited doublet and quartet states were calculated based on g_T and g_R.

Compound	g_T	g_R	g_{D_1}	g_Q	D_T / MHz	D_Q / MHz
H_2TPP-trityl	2.0030	2.0028	2.0031	2.0029	1150	340
ZnTPP-trityl	2.000	2.0028	1.999	2.001	930	310
MgTPP-trityl	2.002	2.0028	2.0017	2.0023	890	260

6.3 Discussion of the triplet contribution to the transient EPR spectra

Transient cw EPR spectra of H_2TPP-trityl and ZnTPP-trityl at different time delays after photoexcitation are shown in Figure S15. In all samples, a minor contribution of the corresponding porphyrin triplet state was observed in addition to the quartet state features. Two possible reasons for this could be imagined: (i) As mentioned above, in a small percentage of the molecules, the trityl radical could have been transformed into the corresponding diamagnetic alcohol. Since the second spin centre would be inactive in this case, a triplet background should be observed in both the optical and EPR experiments. (ii) An alternative reason could originate from a relatively large spread of molecular conformations, frozen in at the freezing point of the solvent: Different molecular conformations – with varying dihedral angles of the phenyl linker – should experience different J-couplings between chromophore and radical. For some conformations, the orbital overlap and thus J_{TR} might be too small for quartet state formation to occur. Such molecules, where the triplet state does not feel the presence of the unpaired electron spin of the radical, would also show up as porphyrin triplets in the transient EPR spectra. Since the spin-polarised EPR signals of porphyrin triplets are very strong, already a contribution of only $\sim$1–3 % could account for the observed triplet background.

To obtain the pure quartet state spectra as shown in the main text, experimental spectra of ZnTPP and H_2TPP were recorded under the same conditions and multiplied by an appropriate scaling factor before subtraction from the corresponding porphyrin-trityl data. Exemplary spectra for ZnTPP-trityl and H_2TPP-trityl before and after subtraction of the triplet signal are shown in Figure S15.

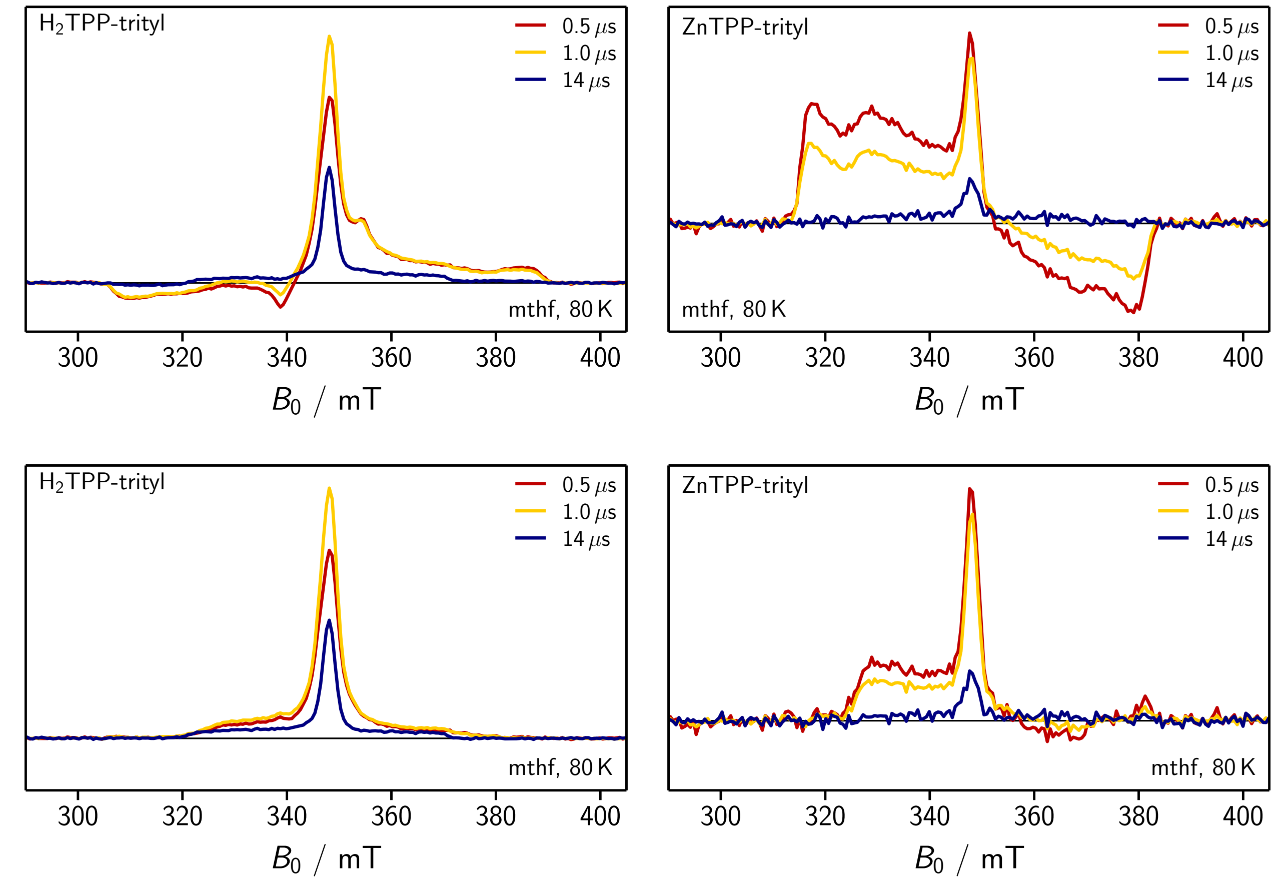

Figure S15: Transient cw EPR spectra of H_2TPP-trityl (*left*) and ZnTPP-trityl (*right*) at different time delays after photoexcitation, before (*top*) and after (*bottom*) subtraction of the triplet background.

6.4 Temperature dependence of the transient EPR spectra

Figure S16 shows transient cw EPR spectra of H_2TPP-trityl and ZnTPP-trityl recorded at different temperatures. It is noted that the spectral shape of the quartet state does not seem to be significantly affected by temperature in the range between 50 and 140 K.

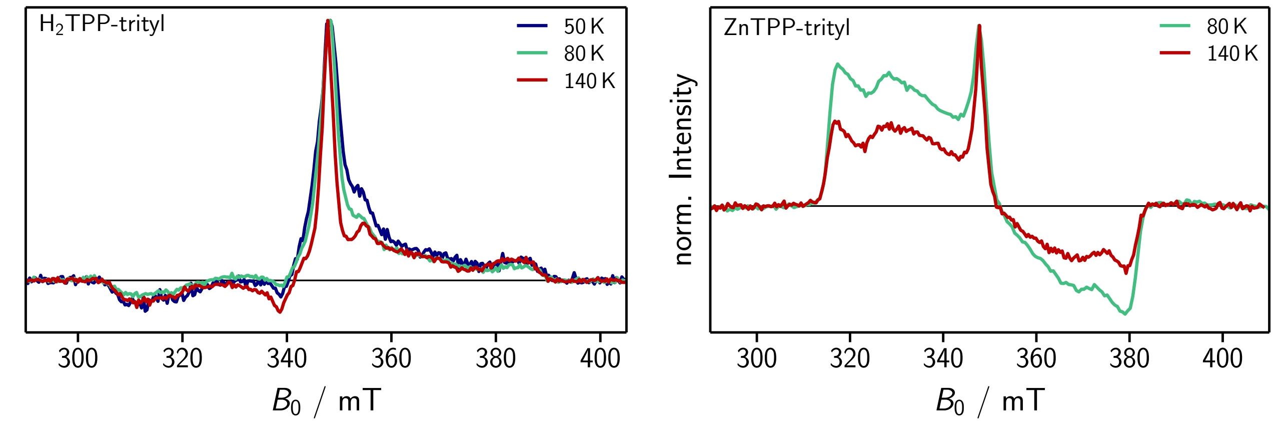

Figure S16: Intensity normalised transient cw EPR spectra of H_2TPP-trityl (*left*) and ZnTPP-trityl (*right*) recorded at different temperatures (as indicated).

6.5 Additional quartet spectra and simulations

Figure S17 shows a schematic energy diagram indicating the allowed EPR transitions of a quartet state. For every orientation of the magnetic field (with respect to the **D**-tensor axes), three transitions are possible, i.e. $|\pm\frac{1}{2}\rangle \leftrightarrow |\pm\frac{3}{2}\rangle$ and $|+\frac{1}{2}\rangle \leftrightarrow |-\frac{1}{2}\rangle$. The maximum spectral separation arises from molecules with their Z-axis parallel to the magnetic field direction and corresponds to $4\,D_Q$.

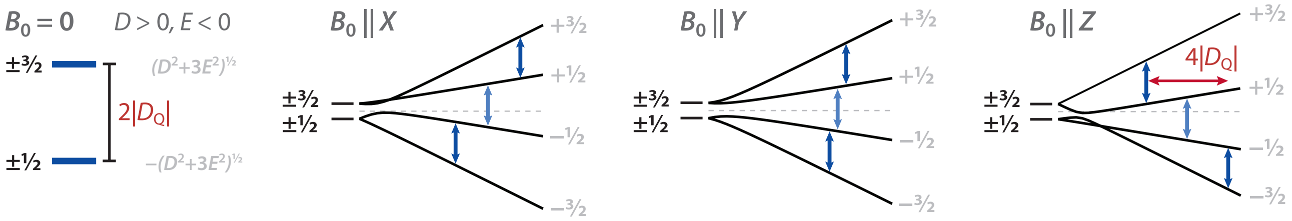

Figure S17: Schematic representation of the energy levels of the quartet state and their Zeeman splitting at different orientations of the external magnetic field with respect to the zero-field-splitting tensor axes. The allowed EPR transitions are indicated by blue arrows.

A comparison of the Q-band transient cw EPR spectra of H_2TPP-trityl and ZnTPP-trityl is shown in Figure S18.

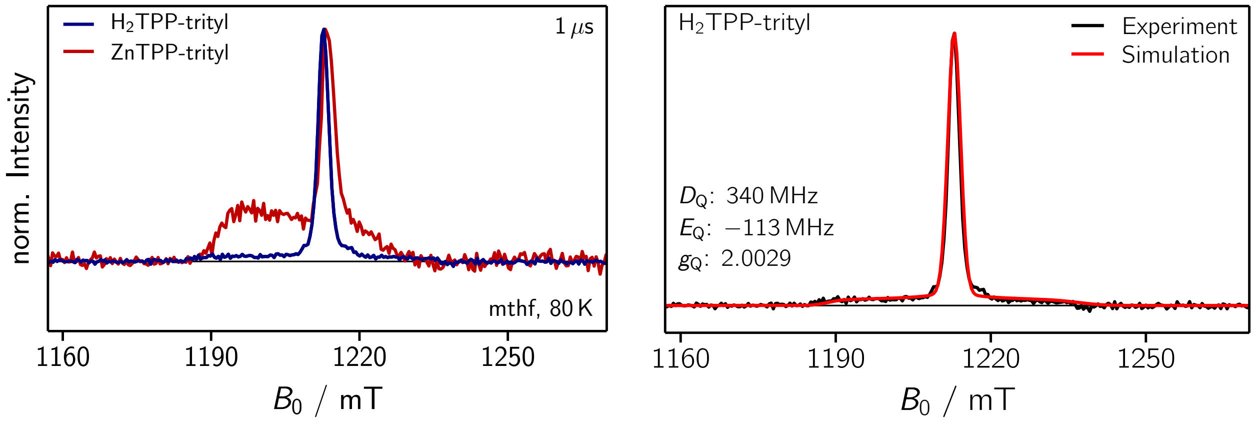

Figure S18: Comparison of the quartet state spectra at $\sim 1\mu$s after photoexcitation of H_2TPP-trityl and ZnTPP-trityl recorded at the Q-band (34.0 GHz) in frozen 2-methyl-THF solution at 80 K (*left*) and simulation of the H_2TPP-trityl spectrum (*right*). The simulation parameters (pure net polarisation) are indicated.

6.6 Discussion of the low signal intensity in transient EPR

Unfortunately it is very difficult to make any predictions about the (expected) strength of spin polarised signals, since the mechanism of spin polarisation cannot be predicted easily. Weak spin polarised EPR signals might either arise from an intrinsically weak spin polarisation (i.e. only small population differences), fast spin relaxation, or the fact that the (potentially strongly) spin polarised species is produced only with a low yield.

Regarding the investigated porphyrin-trityl compounds, it is likely that the weak quartet signal observed by transient cw EPR is due to a low quartet formation yield, since no quartet signal could at all be detected in pulse mode (not even at temperatures as low as 5 K). This interpretation is also in agreement with the optical experiments, suggesting rapid and very efficient deactivation of the chromophore's excited state by energy transfer. In addition, no significant changes in the intensity of the radical signal could be observed

in pulse mode upon photoexcitation at 80 K (cf. Figure S19), also implying a rather low quartet formation yield (assuming that the excitation efficiency is high).

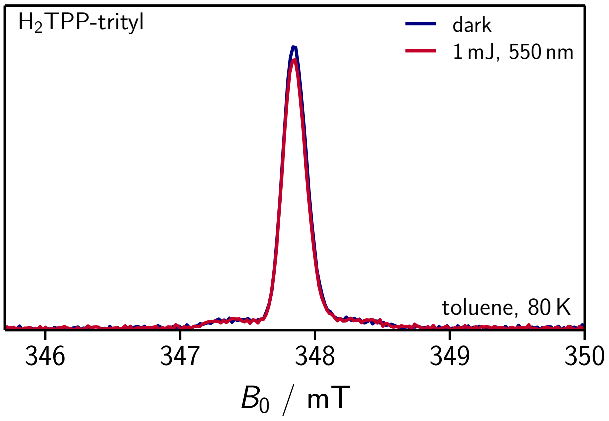

Figure S19: Comparison of the FID-detected field-swept EPR spectra of H_2TPP-trityl recorded with and without photoexcitation in frozen toluene at 80 K.

7 DFT calculations

All DFT calculations were performed using the ORCA program package (version 4.0). For the calculation of the spin density, the structures were first optimised in their (singlet or doublet) ground states using different functionals (CAM-B3LYP, B3LYP, BP86) in combination with the def2-TZVP basis set, RI approximation, and dispersion correction to the energies (D3). Magnetic property calculations (spin densities and hyperfine coupling tensors) in the triplet or quartet states used either the B3LYP or BP86 functionals, in combination with the EPR-II basis set.

For the calculation of the rotational barrier using different functionals (CAM-B3LYP, B3LYP, BP86) in combination with the def2-TZVP basis set, a transition state search and optimisation was first carried out. Frequency calculations confirmed the presence of exactly one imaginary frequency. Starting from the transition state, a series of constraint geometry optimisation steps was performed, scanning the dihedral angle between the porphyrin plane and the plane of the phenyl substituent. Finally, for different thermally accessible dihedral angles, the triplet state spin density was calculated as described above.

A visual representation of some of these results is shown in Figure S20.

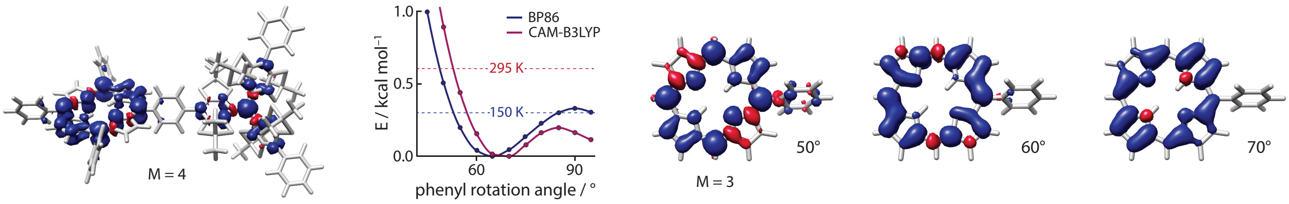

Figure S20: Visualisation of the spin density in the quartet state of H_2TPP-trityl (*left*) and triplet state spin-density distributions on the porphyrin moiety for different thermally accessible dihedral angles between the porphyrin plane and the phenyl linker (*right*).

The different functionals used here predict the energetic minimum for slightly different dihedral angles. However, it can be stated that the energetically preferred dihedral angle is roughly equal to 70°. At room temperature, all dihedral angles between 50 and 130° can be assumed to be significantly populated.

In low temperature measurements, the range of accessible conformations will be governed by the respective situation at the freezing point of the solvent. The freezing point of toluene amounts to roughly 180 K, while that of 2-methyl-THF is ~ 110 K. The exchange interaction between triplet and radical, J_{TR}, will necessarily be different for different molecular conformations since the orbital overlap is affected by rotation of the linker. It is therefore expected that the distribution of exchange interactions is significantly narrower in frozen solution, as compared to room temperature, and shifted towards lower values. The reduced conformational space in frozen solution (restricting the phenyl group rotation to less favourable conformations in terms of orbital overlap) should lead to an overall decrease in J_{TR}, while in fluid solution also conformations with greater $\pi - \pi$ overlap can be accessed.

From the visualisation of the porphyrin triplet state spin density for different dihedral angles of the phenyl linker in Figure S20, it can be seen that already at a dihedral angle of 50° a significant amount of the spin density is spread out onto the phenyl linker.

From the optimised structure of ZnTPP-trityl, a center-to-center distance of 1.3 nm (as used in the calculations of the Förster energy transfer rate) was obtained. However, the effective coupling distance between the two spin centres is predicted to be considerably shorter than the center-to-center distance, due to a significant delocalisation of both the radical and triplet spin densities.

References

[1] Fleck, N.; Hett, T.; Brode, J.; Meyer, A.; Richert, S.; Schiemann, O. C−C cross coupling reactions of trityl radicals: Spin density delocalization, exchange coupling and a spin label. *J. Org. Chem.* **2019**, *84*, 3293–3303.

[2] Lindsey, J. S.; Woodford, J. N. A simple method for preparing magnesium porphyrins. *Inorg. Chem.* **1995**, *34*, 1063–1069.

[3] Taniguchi, M.; Lindsey, J. S. Database of absorption and fluorescence spectra of >300 common compounds for use in PhotochemCAD. *Photochem. Photobiol.* **2018**, *94*, 290–327.

[4] Harriman, A. Luminescence of porphyrins and metalloporphyrins. Part 1.−zinc(II), nickel(II) and manganese(II) porphyrins. *J. Chem. Soc., Faraday Trans. 1* **1980**, *76*, 1978–1985.

[5] Harriman, A. Luminescence of porphyrins and metalloporphyrins. Part 3.−heavy atom effects. *J. Chem. Soc., Faraday Trans. 2* **1981**, *77*, 1281–1291.

[6] Barnett, G. H.; Hudson, M. F.; Smith, K. M. Concerning *meso*-tetraphenylporphyrin purification. *J. Chem. Soc., Perkin Trans. 1* **1975**, 1401–1403.

[7] Montalti, M.; Credi, A.; Prodi, L.; Gandolfi, M. T. *Handbook of photochemistry*; CRC Press, Taylor & Francis Group: Boca Raton, 2006.

[8] Lakowicz, J. R. *Principles of fluorescence spectroscopy*; Springer: New York, 2006.

[9] Laimgruber, S.; Schachenmayr, H.; Schmidt, B.; Zinth, W.; Gilch, P. A femtosecond stimulated raman spectrograph for the near ultraviolet. *Appl. Phys. B* **2006**, *85*, 557–564.

[10] Laimgruber, S.; Schmierer, T.; Gilch, P.; Kiewisch, K.; Neugebauer, J. The ketene intermediate in the photochemistry of *ortho*-nitrobenzaldehyde. *Phys. Chem. Chem. Phys.* **2008**, *10*, 3872–3882.

[11] Fröbel, S.; Buschhaus, L.; Villnow, T.; Weingart, O.; Gilch, P. The photoformation of a phthalide: a ketene intermediate traced by FSRS. *Phys. Chem. Chem. Phys.* **2015**, *17*, 376–386.

[12] Reiffers, A.; Torres Ziegenbein, C.; Schubert, L.; Diekmann, J.; Thom, K. A.; Kühnemuth, R.; Griesbeck, A.; Weingart, O.; Gilch, P. On the large apparent Stokes shift of phthalimides. *Phys. Chem. Chem. Phys.* **2019**, *21*, 4839–4853.

[13] Lorenc, M.; Ziolek, M.; Naskrecki, R.; Karolczak, J.; Kubicki, J.; Maciejewski, A. Artifacts in femtosecond transient absorption spectroscopy. *Appl. Phys. B* **2002**, *74*, 19–27.

[14] Satzger, H.; Zinth, W. Visualization of transient absorption dynamics – towards a qualitative view of complex reaction kinetics. *Chem. Phys.* **2003**, *295*, 287–295.

[15] Baskin, J. S.; Yu, H.-Z.; Zewail, A. H. Ultrafast dynamics of porphyrins in the condensed phase: I. Free base tetraphenylporphyrin. *J. Phys. Chem. A* **2002**, *106*, 9837–9844.

[16] Yu, H.-Z.; Baskin, J. S.; Zewail, A. H. Ultrafast dynamics of porphyrins in the condensed phase: II. Zinc tetraphenylporphyrin. *J. Phys. Chem. A* **2002**, *106*, 9845–9854.

[17] Abraham, B.; Nieto-Pescador, J.; Gundlach, L. Ultrafast relaxation dynamics of photoexcited zinc-porphyrin: Electronic-vibrational coupling. *J. Phys. Chem. Lett.* **2016**, *7*, 3151–3156.

[18] Herb, K.; Tschaggelar, R.; Denninger, G.; Jeschke, G. Double resonance calibration of g factor standards: Carbon fibers as a high precision standard. *J. Magn. Reson.* **2018**, *289*, 100–106.

[19] Stoll, S.; Schweiger, A. EasySpin, a comprehensive software package for spectral simulation and analysis in EPR. *J. Magn. Reson.* **2006**, *178*, 42–55.

[20] Richert, S.; Tait, C. E.; Timmel, C. R. Delocalisation of photoexcited triplet states probed by transient EPR and hyperfine spectroscopy. *J. Magn. Reson.* **2017**, *280*, 103–116.

[21] Tait, C. E.; Neuhaus, P.; Anderson, H. L.; Timmel, C. R. Triplet state delocalization in a conjugated porphyrin dimer probed by transient electron paramagnetic resonance techniques. *J. Am. Chem. Soc.* **2015**, *137*, 6670–6679.

[22] Van der Waals, J. H.; Van Dorp, W. G.; Schaafsma, T. J. In *The Porphyrins, Vol IV*; Dolphin, D., Ed.; Academic Press Inc.: New York, 1979.

[23] Tait, C. E.; Neuhaus, P.; Peeks, M. D.; Anderson, H. L.; Timmel, C. R. Transient EPR reveals triplet state delocalization in a series of cyclic and linear pi-conjugated porphyrin oligomers. *J. Am. Chem. Soc.* **2015**, *137*, 8284–8293.
